# Rational Design and Structure–Activity Relationship Analysis of Decalin-Based High-Energy-Density Fuel Molecules

**DOI:** 10.3390/molecules31162911

**Published:** 2026-08-20

**Authors:** Ya-Ling Gong, Wen-Ying Li

**Affiliations:** State Key Laboratory of Clean and Efficient Coal Utilization, Taiyuan University of Technology, No. 79 Yingze West Street, Taiyuan 030024, China; gongyaling0172@link.tyut.edu.cn

**Keywords:** coal-to-liquid, polycyclic hydrocarbons, fuel molecular design, quantitative structure–property relationship, multi-criteria screening

## Abstract

Decalin, accessible through the hydrogenation of coal-tar-derived naphthalene, is a saturated bicyclic hydrocarbon consisting of two fused cyclohexane rings, and provides a promising platform for high-energy-density fuel (HEDF) design. A systematic library of alkyl- and cycloalkyl-substituted *trans*-decalin derivatives was constructed to investigate structure–property relationships involving density, net heat of combustion (NHOC), specific impulse, viscosity, flash point, and thermal/oxidative stability. Minimum C–C and C–H bond dissociation enthalpies were used to comparatively assess thermal and oxidative stability by reflecting initial C–C homolysis and H abstraction tendencies, respectively. Density and volumetric NHOC were governed mainly by molecular compactness and packing efficiency, whereas gravimetric NHOC and specific impulse depended primarily on the H/C ratio and ring strain. The flash point was mainly associated with molecular mass, while viscosity was influenced by molecular mass and molecular architecture. Under the engineering-oriented constraints of viscosity ≤ 16 mPa·s and flash points ≥ 360 K, multi-objective screening identified the spiro four-membered-ring motif, particularly α-S-Cycle4, as providing the best overall property balance. In contrast, the fused three-membered-ring motif showed higher specific impulse but greater susceptibility to initial bond activation, suggesting its potential use as a blending component. This work provides quantitative structure-based guidelines for the rational design of HEDFs.

## 1. Introduction

High-energy-density fuels (HEDFs) are critical for advanced aerospace propulsion systems, as their high energy content can extend the vehicle range and increase the payload capacity [1,2,3]. Desirable HEDFs must combine high gravimetric and volumetric net heat of combustion (NHOC) with acceptable low-temperature fluidity, handling safety, and thermal/oxidative stability [2,3]. For rocket propulsion, a higher specific impulse reduces the propellant mass required to deliver a given total impulse [4]. However, these properties are governed by distinct and sometimes competing structural factors, making their simultaneous optimization challenging in rational HEDF design [1,3].

Conventional HEDFs (e.g., JP-10, RJ-7) are predominantly petroleum-derived polycyclic hydrocarbons with high volumetric energy density and favorable combustion characteristics [1,2]. Coal tar, a byproduct of coal coking, is a potential non-petroleum feedstock because it is rich in PAHs that can be hydrogenated to saturated polycyclic hydrocarbons [5,6]. Their compact multi-ring structures generally favor high liquid density and volumetric energy density [2,3]. Naphthalene is typically the most abundant individual PAH in high-temperature coal tar, possesses a well-defined C10 fused bicyclic framework, and can be separated and purified using established industrial processes [5,7]. Complete hydrogenation of naphthalene produces decalin [8]. Decalin is an important cycloalkane component of JP-900, a thermally stable, high-density fuel developed through a coal-based route [6]. Given its well-defined saturated bicyclic structure and potential for further modification, decalin was therefore selected as the parent scaffold for designing substituted HEDF candidates.

Alkyl substitution and branching can improve the low-temperature behavior of rigid cyclic and cage hydrocarbons while retaining a relatively high density [9,10]. Strained cyclopropane and cyclobutane units have been introduced to increase energy content through ring-strain energy [11,12,13]. Studies of spirocyclic and other compact polycyclic hydrocarbons have further indicated that ring connectivity can influence fuel-relevant properties, although general topology–property relationships remain unclear [14,15]. For decalin-based fuels, experimental studies have mainly focused on selected branched or alkylated products and product mixtures [9,16]. For alkyl- and cycloalkyl-substituted decalin derivatives, systematic experimental data covering multiple fuel properties remain fragmented or unavailable. Given the extensive synthesis, purification, and characterization required for a controlled experimental series, a virtual library provides a practical basis for systematic and internally consistent comparison.

Quantum-chemical calculations and molecular dynamics have been combined to predict combustion enthalpy, density, and related properties for selected candidates [17]. A database of 178 cycloalkanes has also been used to derive broad relationships between the molecular structure, density, and energy content [15]. Machine-learning methods have been applied to high-throughput screening of large hydrocarbon libraries [18]. Statistical models have been used to evaluate selected fuel candidates [19], while database-guided substructure mining has been used to generate new HEDF candidates [20]. However, these studies have generally examined either selected candidates or molecules with different parent scaffolds, making it difficult to determine the effects of individual structural factors. No systematic decalin library has been reported in which the substitution position, alkyl chain length and branching, cycloalkyl ring size, and connection topology are varied in a controlled manner. Consequently, the effects of these factors on individual fuel properties and the trade-offs among multiple properties remain insufficiently understood.

In this work, a rationally designed library of alkyl- and cycloalkyl-substituted *trans*-decalin derivatives was constructed by varying the substitution position, alkyl chain length and branching, cycloalkyl ring size, and connection topology. An integrated computational workflow was used to evaluate the density, gravimetric and volumetric NHOC, specific impulse, low-temperature viscosity, and flash point. Minimum C–C and C–H bond dissociation energies were used as thermodynamic descriptors of susceptibility to initial C–C homolysis and H abstraction relevant to thermal and oxidative degradation, respectively. Multiple linear regression quantified the contributions of individual structural variables and established the interpretable structure–property relationships, while multi-objective screening evaluated the trade-offs among key fuel properties and identified the candidates with balanced performance. By integrating property prediction, interpretable regression, and multi-objective screening within a scaffold-controlled molecular library, this work provides quantitative guidance for decalin-based HEDF design.

## 2. Results and Discussion

### 2.1. Density and Volumetric Energy Density

Density and volumetric NHOC are core macroscopic metrics for evaluating the volume-based energy performance of HEDFs. The calculated density and volumetric NHOC values vary considerably across the designed decalin derivatives, indicating that both properties are sensitive to substituent architecture.

**Figure 1:**
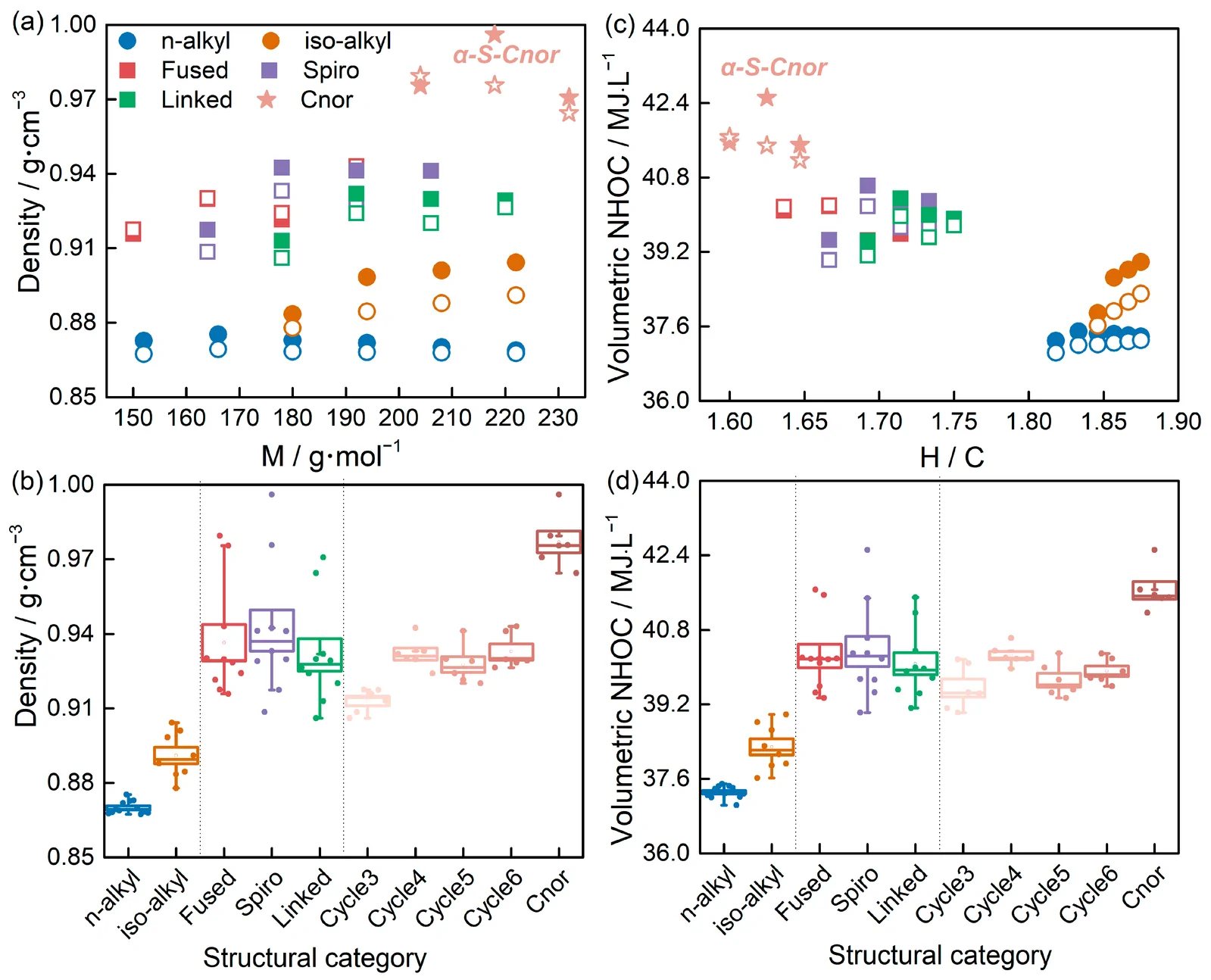
Density and volumetric NHOC of substituted *trans*-decalin derivatives. (**a**) Density as a function of molar mass. (**b**) Density distributions grouped by structural category. (**c**) Volumetric NHOC as a function of the H/C ratio. (**d**) Volumetric NHOC distributions by structural category. Filled and open symbols denote α- and β-substituted derivatives, respectively. For boxplots, boxes indicate interquartile ranges, horizontal lines indicate medians, whiskers show data spread, and points represent individual molecules.

As shown in Figure 1a, alkyl- and cycloalkyl-substituted derivatives display distinct density trends. For n-alkyl derivatives, density shows only a shallow maximum at C2 and then decreases slightly with further chain extension, whereas iso-alkyl derivatives are generally denser than their n-alkyl isomers. The positional effect is weak, with α-substituted derivatives showing slightly higher densities than the corresponding β-substituted isomers. For cycloalkyl-substituted derivatives, density shows no simple mass-dependent trend, and the scatter among structures with similar molar masses indicates that molar mass alone cannot explain the observed variations. The grouped distributions in Figure 1b further show that cycloalkyl-substituted derivatives occupy a higher density region than alkyl-substituted derivatives. Linked structures are mainly distributed in the lower density region, whereas fused and spiro structures shift toward higher densities. Among the different ring types, Cycle3 derivatives give relatively low densities, while Cnor derivatives show the highest values. Cycle4–Cycle6 derivatives fall in an intermediate and overlapping density range, with the Cycle4 and Cycle6 derivatives being slightly denser than Cycle5 derivatives on average. Notably, α-S-Cnor gives the highest density in the present molecular library, indicating that the combination of α-substitution, spiro topology, and the norbornyl-type ring is particularly favorable for density enhancement.

Volumetric NHOC was further compared as a density-related volume-based energy property. As shown in Figure 1c, volumetric NHOC does not simply follow the H/C ratio. Although alkyl-substituted derivatives have relatively high H/C ratios, they remain in the lower volumetric energy range and show only limited variation. By contrast, cycloalkyl-substituted derivatives display a broader distribution and generally give larger heat release per unit volume. The grouped distributions in Figure 1d show a trend broadly consistent with the density results. Cnor derivatives give the largest volumetric heat release, whereas Cycle3 derivatives remain in the lower range. Overall, the structural classes favorable for high density also tend to exhibit a higher volumetric energy density, with α-S-Cnor representing the most prominent example of simultaneous density and volumetric NHOC enhancement.

To express density in a mass-normalized volume form and facilitate the quantitative comparison of structural effects, V_m_/M, which is equal to 1/ρ, was adopted as the dependent variable. A lower V_m_/M therefore corresponds to a higher density. The MLR model included the alkyl chain length (N_C_), substitution position (I_α_), branching (I_iso_), cycloalkyl ring type (I_Cycle3_, I_Cycle4_, I_Cycle5_, I_Cycle6_, I_Cnor_), and connection topology (I_Fused_, I_Spiro_), with β-n-C1 and linked topology used as reference levels. The descriptor definitions, regression coefficients, model-performance metrics, residual analysis, outlier identification, and leverage-based applicability-domain assessment are provided in Tables S2.2 and S2.3 and Figure S2.1 of the Supplementary Materials.

The resultant regression equation is expressed below:

$$V_{m}/M\;=\;1.155\;-\;0.001N_{C}\;-\;0.007I_{\alpha }\;-\;0.026I_{\mathrm{iso}}\;-\;0.051I_{\mathrm{Cycle}3}\;-\;0.073I_{\mathrm{Cycle}4}$$

$$-0.068I_{\mathrm{Cycle}5}-0.074I_{\mathrm{Cycle}6}-0.123I_{\mathrm{Cnor}}-0.006I_{\mathrm{Fused}}-0.011I_{\mathrm{Spiro}}$$

**Figure 2:**
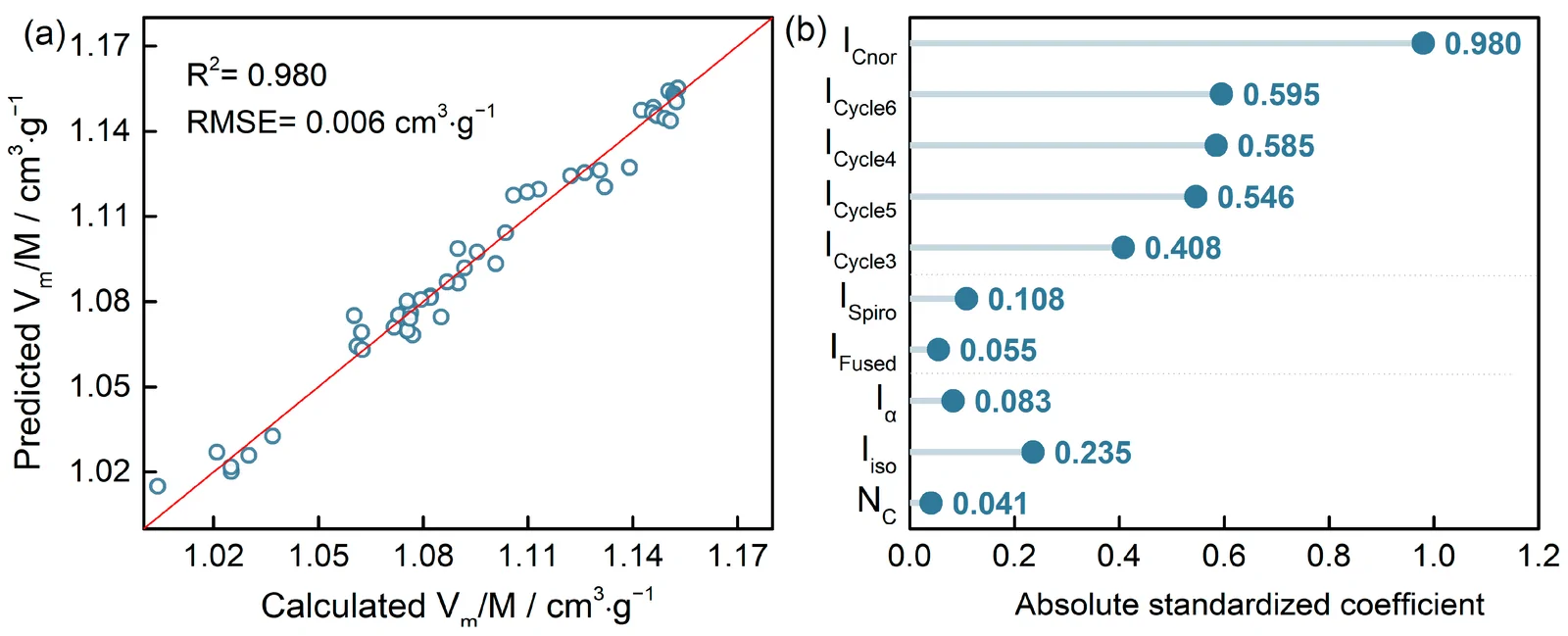
V_m_/M model performance and descriptor effects. (**a**) Predicted versus calculated V_m_/M. The red line represents y = x. (**b**) Absolute standardized regression coefficients; larger values indicate stronger density-favorable effects.

The model exhibits excellent performance, with adjusted R^2^ = 0.975, Q^2^ = 0.967, and RMSE = 0.006 cm^3^·g^−1^. As illustrated in Figure 2a, the predicted V_m_/M values agree well with the calculated values across the molecular library, indicating that the selected descriptors capture the main density-related structural variations. The standardized descriptor effects in Figure 2b are discussed in terms of density enhancement. For alkyl substituents, I_iso_ contributes more strongly than N_C_, indicating that branching is more effective than linear chain extension in reducing V_m_/M and thus favoring a higher density. I_α_ gives a smaller contribution, which is consistent with the weak positional effect observed in Figure 1. For cycloalkyl-substituted derivatives, I_Cnor_ gives the largest density-favorable contribution, followed by I_Cycle6_ and I_Cycle4_, whereas I_Cycle3_ shows the weakest effect. Therefore, the ring-type contribution follows the order Cnor ≫ Cycle6 ≈ Cycle4 > Cycle5 > Cycle3. Among the topology descriptors, I_Spiro_ shows a significant density-favorable contribution relative to the linked reference, whereas the I_Fused_ coefficient is smaller and marginally nonsignificant. The estimated coefficients therefore suggest an overall density-favorable tendency of spiro > fused > linked, although the fused topology difference is not clearly resolved at the conventional 0.05 significance level.

To clarify the microscopic origin of density variations, V_m_/M was decomposed into mass-normalized intrinsic and free volume contributions, V_int_/M and V_free_/M [21]. V_int_ represents the intrinsic occupied volume of an individual molecule, reflecting intrinsic molecular compactness [22], whereas V_free_ reflects the intermolecular free volume associated with liquid packing [23]. R_g_ and χ_asph_ denote the radius of gyration and molecular asphericity, reflecting the molecular size and shape anisotropy, respectively, while ln(N_conf_+1) and σ(V) describe conformational accessibility and conformational volume dispersion. The correlations with V_int_ and V_free_ are provided in Figure S2.2 of the Supplementary Materials. These microscopic descriptors were used to rationalize the MLR-identified density-favorable factors.

**Figure 3:**
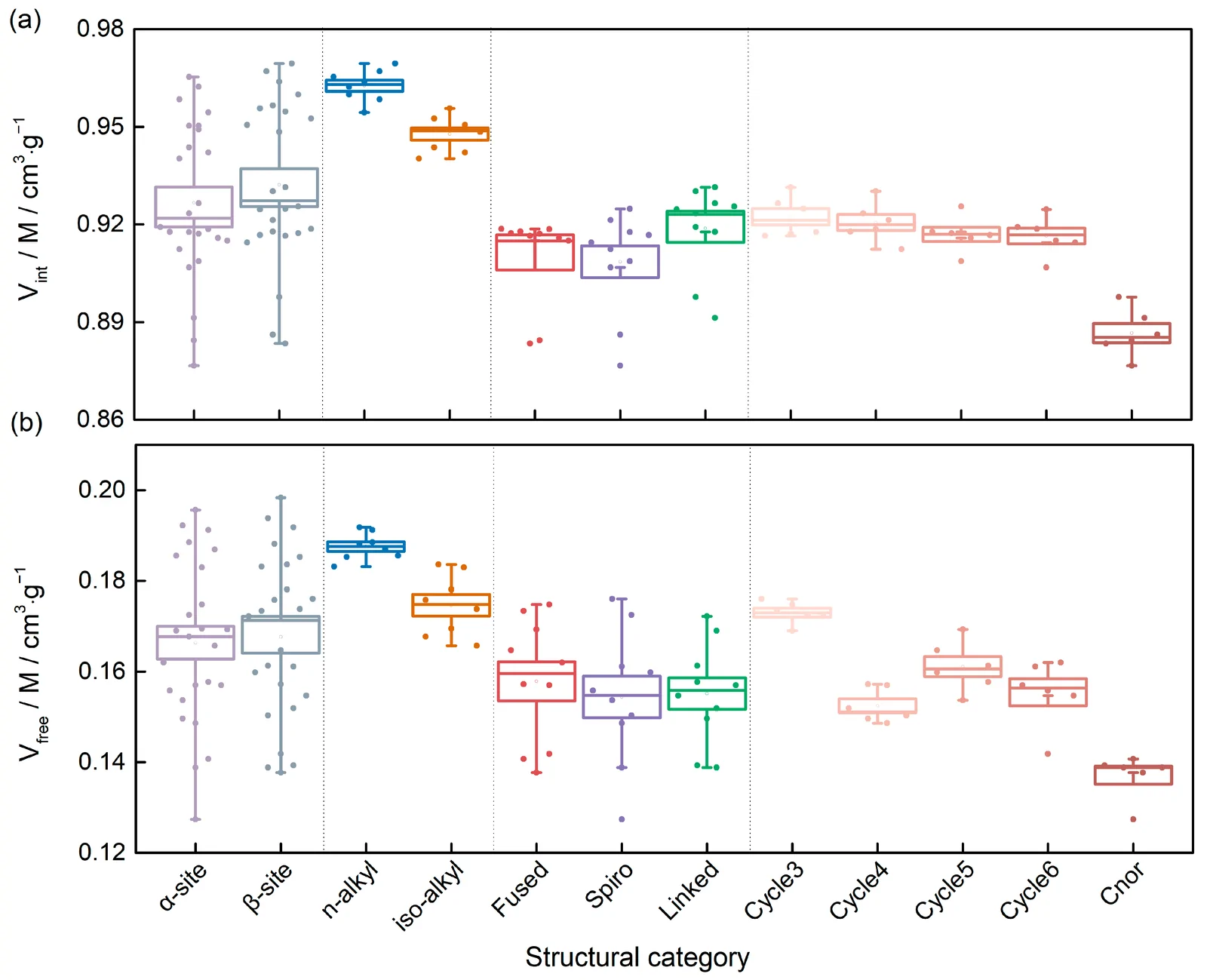
Mass-normalized intrinsic and free volume components of substituted *trans*-decalin derivatives. (**a**) V_int_/M and (**b**) V_free_/M distributions grouped by structural category. The boxplot notations are consistent with those used in Figure 1.

As shown in Figure 3, the α-site lies closer to the ring junction of the *trans*-decalin scaffold than the β-site and slightly suppresses outward extension, as reflected by the modest decreases in R_g_ and χ_asph_. However, positional isomerism does not alter the branching mode or number of rotatable bonds, so its influence on ln(N_conf_+1) and σ(V) is limited. Consequently, α-substitution only slightly reduces both V_int_/M and V_free_/M, leading to a weak density increase. Consistent with the stronger I_iso_ contribution in the MLR model, branching gives a more pronounced effect. Iso-alkyl substitution interrupts continuous chain extension and reduces conformational freedom, giving lower R_g_, χ_asph_, ln(N_conf_+1), and σ(V). These changes are consistent with reduced V_int_/M and V_free_/M contributions, explaining the higher densities of iso-alkyl derivatives.

Among cycloalkyl-substituted derivatives, in line with the largest I_Cnor_ contribution in the MLR model, Cnor shows the most substantial reduction in V_m_/M, consistent with its rigid bridged structure, which limits molecular extension and restricts conformational variation, as reflected by the lower mass-corrected residuals of R_g_, χ_asph_,ln(N_conf_+1), and σ(V). These descriptor changes jointly support the reductions in V_int_/M and V_free_/M. For ordinary cycloalkyl substituents, variations in V_m_/M are mainly governed by V_free_/M. Cycle3 derivatives retain relatively high V_free_/M, suggesting that their locally constrained triangular geometry is less favorable for efficient intermolecular packing. In contrast, Cycle4 derivatives show lower V_free_/M, which may be associated with their nonplanar ring geometry and improved packing compatibility. Cycle5 derivatives are less favorable because their higher mass-corrected residuals of ln(N_conf_+1) and σ(V) indicate broader conformational accessibility and larger conformational volume dispersion, thereby increasing packing heterogeneity. Cycle6 derivatives also show relatively low V_free_/M, consistent with a more regular low-energy, nonplanar conformation and smaller conformational volume dispersion. Connection topology also modulates molecular density. The linked topology connects the substituent ring to the *trans*-decalin scaffold through an external single bond, allowing the ring to extend farther away from the core. This is reflected by the higher mass-corrected residuals of R_g_, χ_asph_, ln(N_conf_+1), and σ(V), indicating stronger molecular extension and broader conformational volume dispersion. These properties make linked topology less favorable for achieving high density. By contrast, the spiro topology keeps the substituent ring closer to a single connection center and reduces outward extension, whereas the fused topology remains intermediate. Therefore, topology changes density by jointly modulating both intrinsic compactness and packing compatibility, following the overall density-favorable tendency of spiro > fused > linked.

The combined analyses show that density is governed by molecular mass and structural factors. For alkyl-substituted derivatives, branching and α-substitution favor higher densities by enhancing molecular compactness and packing efficiency, whereas simple linear chain extension has a relatively limited effect. For cycloalkyl-substituted derivatives, compact ring structures and spiro topology are more favorable for density enhancement. Volumetric NHOC exhibits similar trends to density, as it depends on both density and gravimetric NHOC. Spiro norbornyl-type and four-membered-ring derivatives show the most favorable volumetric energy density, and may therefore be favorable for volume-limited aerospace applications.

### 2.2. Gravimetric Energy Density and Specific Impulse

Gravimetric NHOC and I_sp_ are key metrics for evaluating mass-based energy and propulsion performance of HEDFs. Gravimetric NHOC represents the heat release per unit mass, while I_sp_ provides a comparative measure of propulsion performance under fixed calculation conditions.

**Figure 4:**
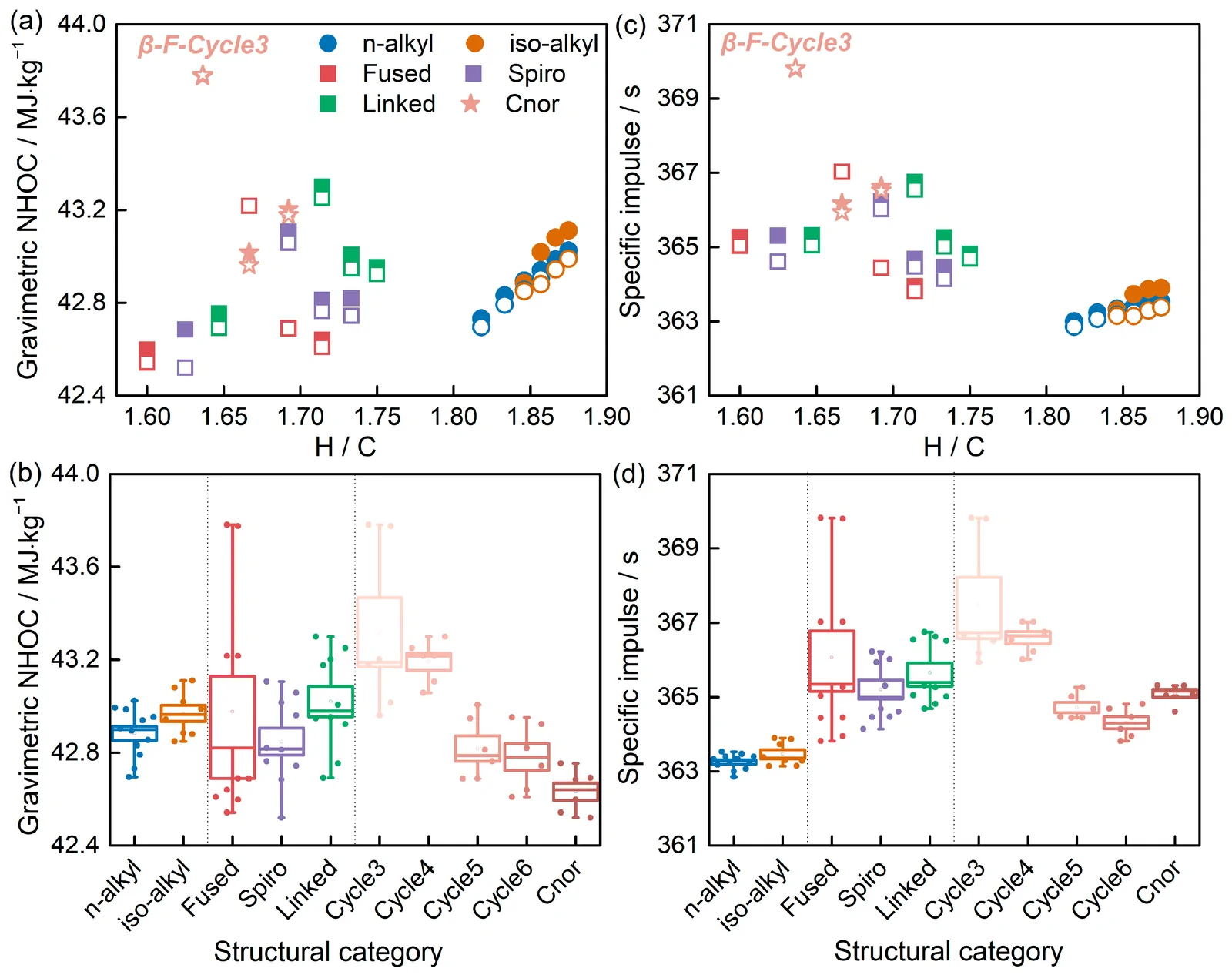
Gravimetric NHOC and specific impulse of substituted *trans*-decalin derivatives. (**a**) Gravimetric NHOC and (**c**) specific impulse as a function of the H/C ratio; (**b**) Gravimetric NHOC and (**d**) specific impulse distributions grouped by structural category. Symbol coding and boxplot notations follow those used in Figure 1.

Gravimetric NHOC varies within a relatively narrow range across the substituted *trans*-decalin derivatives. As shown in Figure 4a, alkyl-substituted derivatives generally show an increase in the heat release per unit mass with an increasing H/C ratio, and α-substituted derivatives are consistently slightly higher than their β-substituted isomers. The effect of branching depends on the substitution position, with iso-alkyl derivatives being slightly favored at the α-site, whereas this effect becomes weaker or less regular at the β-site. For cycloalkyl-substituted derivatives, gravimetric NHOC does not show a simple monotonic dependence on the H/C ratio, and a noticeable dispersion remains among molecules with similar compositions. The grouped distributions in Figure 4b further show that fused, spiro, and linked structures largely overlap, with linked structures exhibiting a slightly higher median value and fused structures showing a broader distribution. In contrast, the ring type gives a more evident distinction, as Cycle3 and Cycle4 derivatives generally show higher gravimetric NHOC values, while Cycle5, Cycle6, and Cnor derivatives shift toward a lower range.

As shown in Figure 4c, the specific impulse exhibits a macroscopic trend broadly consistent with gravimetric NHOC. Alkyl-substituted derivatives show a gradual increase in specific impulse with the H/C ratio, whereas cycloalkyl-substituted derivatives display a broader distribution and generally higher specific impulse. In the grouped distributions, fused, spiro, and linked structures show substantial overlap, indicating that topology is not the dominant factor for specific impulse at the macroscopic level. The ring type gives a more distinct variation, as Cycle3 derivatives exhibit the highest specific impulse, followed by Cycle4 derivatives, while Cycle5 and Cycle6 derivatives remain in the lower range. Cnor derivatives occupy an intermediate region. In summary, gravimetric NHOC and specific impulse are mainly associated with molecular composition, while the ring type introduces additional structural modulation beyond the H/C ratio. Notably, β-F-Cycle3 represents the most prominent example of the simultaneous enhancement of gravimetric NHOC and specific impulse.

To quantitatively resolve the structure–property relationships inferred from the macroscopic analysis and separate compositional, strain-related, and topology-related contributions, MLR models were constructed for gravimetric NHOC and I_sp_ using physically interpretable descriptors. The H/C ratio was introduced as the primary compositional descriptor, while ring-strain energy (E_strain_) was added to represent ring-strain effects. I_Fused_, I_Spiro_, I_Linked_, I_iso_, and I_α_ were introduced to characterize the connection topology, branching, and substitution position. In addition, the fused–cyclopropane interaction descriptor E_strain_ × I_Fused_ × I_Cycle3_ was included to describe the localized coupling between the cyclopropane ring strain and fused topology. The regression equations are given below, and the descriptor definitions, regression coefficients, model-performance metrics, residual analysis, outlier identification, and leverage-based applicability-domain assessment are summarized in Tables S3.2–S3.7 and Figures S3.1 and S3.2 of the Supplementary Materials.

$$Gravimetric\;\mathrm{NHOC}=33.625+4.992(H/C)+0.054I_{\alpha }+0.027I_{\mathrm{iso}}+0.511I_{\mathrm{Fused}}+0.426I_{\mathrm{Spiro}}$$

$$+0.493I_{\mathrm{Linked}}+0.023E_{\mathrm{strain}\;}+0.030E_{\mathrm{strain}}\;\times \;I_{\mathrm{Fused}}\;\times \;I_{\mathrm{Cycle}3}$$

(1) $$I_{sp}=348.043+8.161(H/C)+0.229I_{\alpha }+0.119I_{\mathrm{iso}\;}+2.140I_{\mathrm{Fused}}+1.778I_{\mathrm{Spiro}}+2.063I_{\mathrm{Linked}}\;+0.126E_{\mathrm{strain}}\;\times \;I_{\mathrm{Fused}}\;\times \;I_{\mathrm{Cycle}3}+0.097E_{\mathrm{strain}}$$

**Figure 5:**
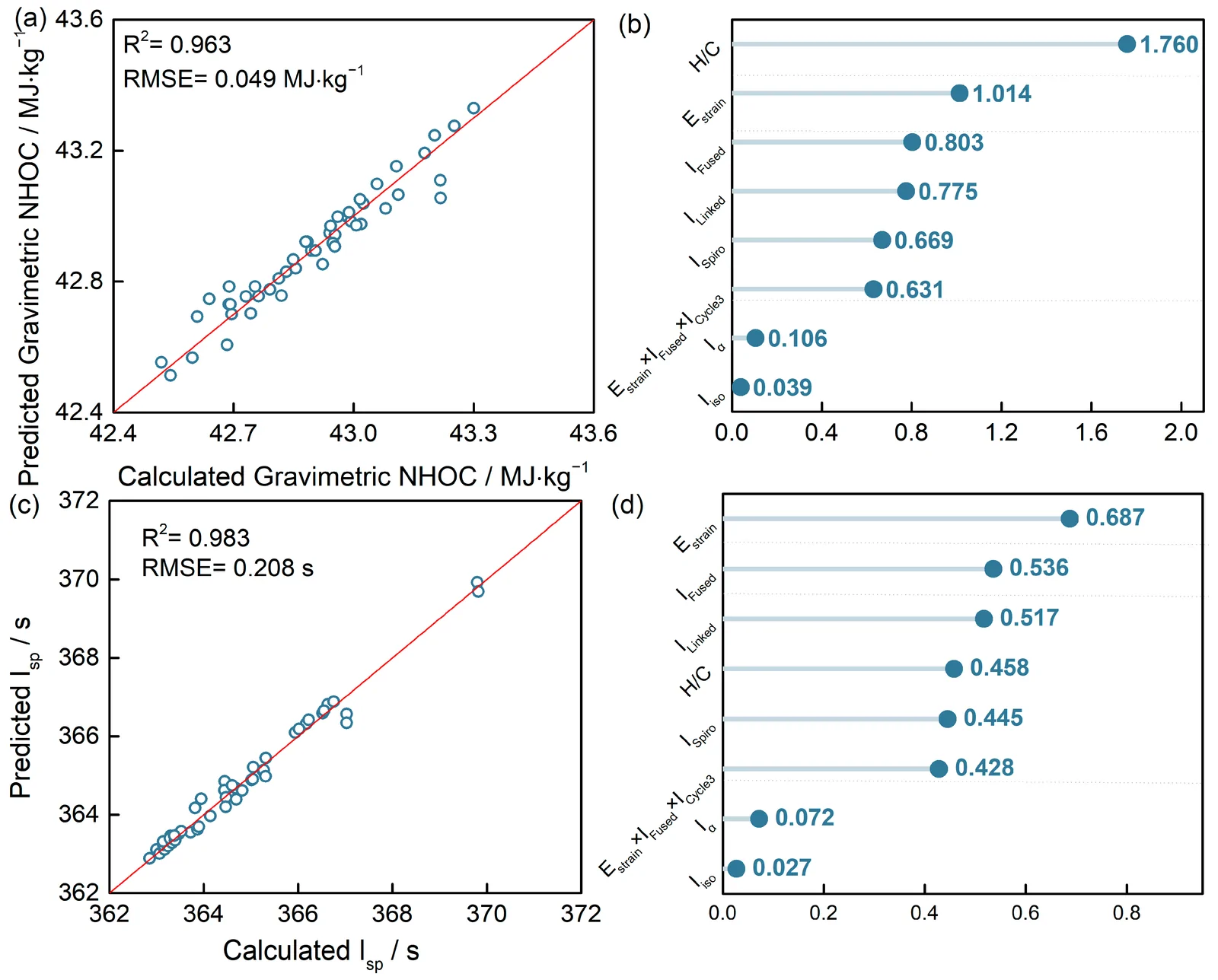
Gravimetric NHOC and I_sp_ model performance and descriptor effects. Predicted versus calculated (**a**) gravimetric NHOC and (**c**) I_sp_. Standardized descriptor effects on (**b**) gravimetric NHOC and (**d**) I_sp._

The gravimetric NHOC model gives an adjusted R^2^ = 0.956, a cross-validated Q^2^ = 0.942, and an RMSE = 0.049 MJ·kg^−1^, indicating good agreement between the predicted and calculated gravimetric NHOC values. The I_sp_ model also showed high predictive reliability, giving an adjusted R^2^ = 0.980, Q^2^ = 0.973, and RMSE = 0.208 s. The close agreement between R^2^ and Q^2^ indicates that the models are not merely fitted to the training dataset but retain stable internal predictive capability. As shown in Figure 5, the predicted values are closely distributed along the y = x line for both gravimetric NHOC and $I_{sp}$, confirming that the selected descriptors effectively capture the dominant structural factors governing mass-based energy release and propulsion performance. The residual and applicability-domain analyses provided in the Supplementary Materials further indicate that no applicability-domain outlier was identified within the investigated decalin-based structural space.

The standardized coefficients further clarify the relative structural contributions to gravimetric NHOC and I_sp_. As shown in Figure 5b, gravimetric NHOC is mainly governed by the H/C ratio, followed by E_strain_ and topology-related descriptors. This indicates that molecular composition defines the baseline mass-based heat release, while the ring strain and connection topology provide additional structural modulation. Among the topology descriptors, fused and linked topologies show stronger positive effects than spiro topology, and the E_strain_ × I_Fused_ × I_Cycle3_ term further reflects the localized strain–topology coupling in fused Cycle3 derivatives. In contrast, Figure 5d shows that I_sp_ is more sensitive to E_strain_, followed by topology-related descriptors and the H/C ratio, indicating that propulsion performance is strongly affected by strain-related structural variation. In both models, I_α_ and I_iso_ show relatively weak contributions, consistent with the minor positional and branching effects observed in the macroscopic trends.

The variations in gravimetric NHOC and I_sp_ can be rationalized in terms of molecular composition and strain-related energetic effects. The H/C ratio defines the compositional baseline because hydrogen releases substantially more energy per unit mass upon oxidation than carbon (142.9 vs. 32.8 kJ·g^−1^) [24]. Consequently, increasing H/C generally favors higher gravimetric NHOC and higher I_sp_. The effect of ring type is represented here through E_strain_. Cycle3 and Cycle4 motifs deviate strongly from the ideal tetrahedral geometry, with average ring C–C–C angles of approximately 60° and 90°, respectively, and therefore retain the greater angular strain of larger cycloalkyl rings [25,26]. Cnor derivatives also contain considerable strain because their rigid bridged framework imposes strong geometric constraints [27]. However, the strain contribution follows the general order Cycle3 > Cycle4 > Cnor > Cycle5 > Cycle6, indicating that the strain-driven enhancement is most pronounced for three- and four-membered rings. Thus, a higher E_strain_ corresponds to a higher intrinsic energy of the molecule, which is consistent with the regression results in which E_strain_ is associated with higher gravimetric NHOC and higher I_sp_. The topology effect may be interpreted as the extent to which different connection modes introduce additional strain at the junction. Fused structures share an edge with the decalin scaffold, which is likely to impose the strongest local geometric constraint. Linked structures are connected through a rotatable C–C single bond, allowing partial conformational adjustment, whereas spiro structures share only one carbon atom and can maintain more independent local geometries [28,29]. This structural interpretation is consistent with the standardized descriptor effects, in which fused topology gives a larger contribution than spiro topology in both gravimetric NHOC and I_sp_ models. By comparison, the substitution position and branching exert only weak effects, as neither changes the compositional baseline defined by H/C nor the ring-type contribution represented by E_strain_. The E_strain_ × I_Fused_ × I_Cycle3_ descriptor further captures the localized strain–topology coupling in fused Cycle3 derivatives, where the severe angular strain of the three-membered ring is combined with the geometric constraint introduced by fused connectivity. This coupled contribution is consistent with the particularly high gravimetric NHOC and $I_{sp}$ of fused Cycle3 derivatives, especially β-F-Cycle3.

Overall, gravimetric NHOC and I_sp_ reflect mass-based energy and propulsion performance, and are closely linked to the H/C ratio and ring strain. For alkyl-substituted derivatives, gravimetric NHOC and I_sp_ increase with the H/C ratio, with weak positional and branching effects. For cycloalkyl-substituted derivatives, strained small rings (Cycle3, Cycle4) significantly enhance gravimetric NHOC and I_sp_ due to the high ring strain. The final MLR models reveal that the H/C ratio gives the largest standardized contribution to gravimetric NHOC, whereas E_strain_ gives the largest standardized contribution to I_sp_. Fused topology and the fused–cyclopropane interaction further enhance both properties through localized strain–topology coupling. Notably, β-F-Cycle3 exhibits the highest gravimetric NHOC and I_sp_, attributed to the combined strain of the fused three-membered ring and its junction with the *trans*-decalin scaffold, highlighting the advantage of this motif for propulsion-related performance.

### 2.3. Viscosity and Flash Point

The viscosity and flash point were used as auxiliary engineering descriptors for evaluating low-temperature transport behaviors and handling safety. The predicted viscosity at 233.15 K ranges from 5.72 to 79.48 mPa·s, while the flash point ranges from 332.61 to 421.43 K, indicating that both properties are sensitive to the molecular structure. Because the viscosity data are strongly skewed, ln(η) is used for the following discussion.

**Figure 6:**
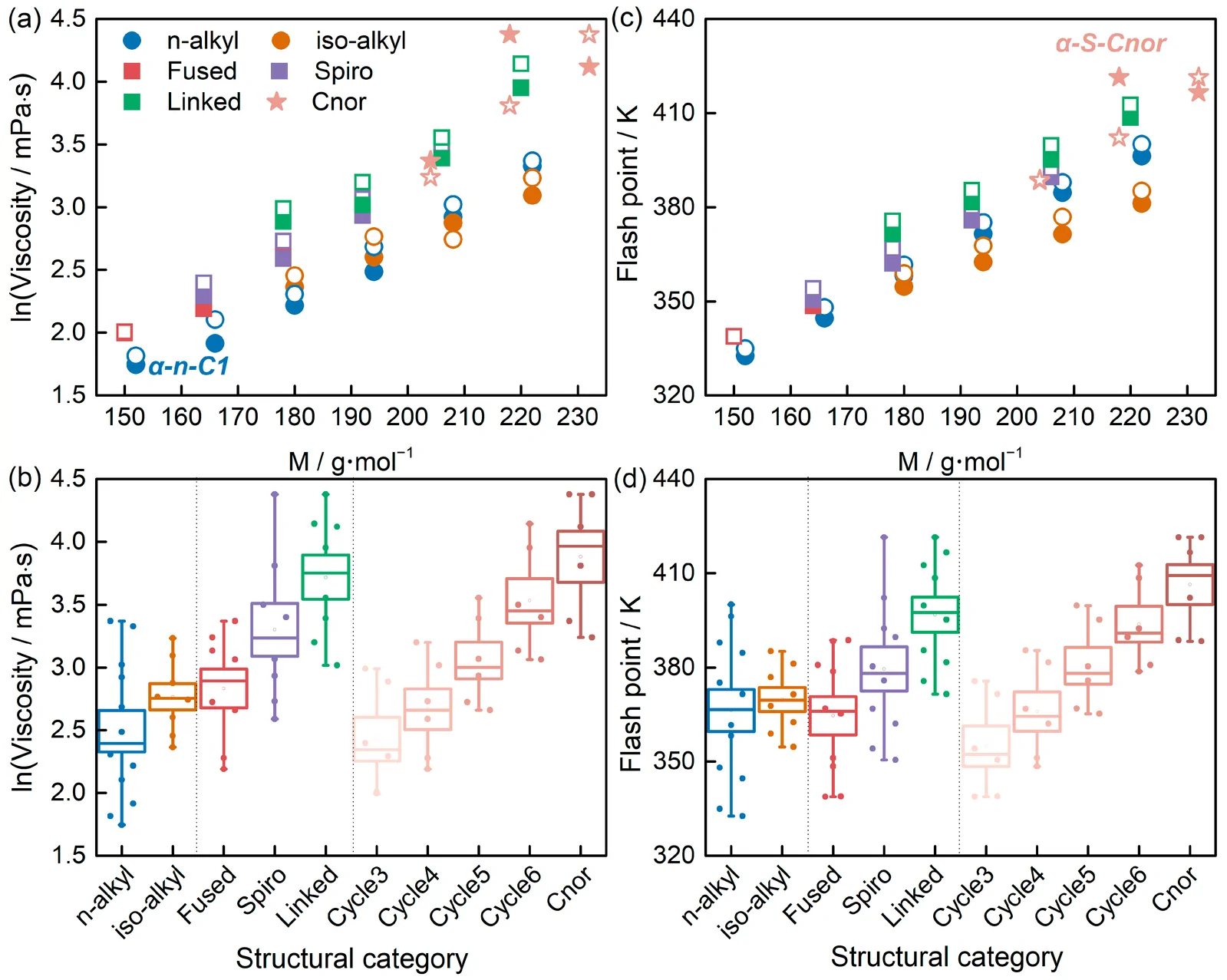
ln(η) and flash points of substituted *trans*-decalin derivatives. (**a**) ln(η) and (**c**) flash points as a function of molar mass. (**b**) ln(η) and (**d**) flash-point distributions grouped by structural category. Symbol coding and boxplot notations follow those used in Figure 1.

As shown in Figure 6a, ln(η) of the alkyl-substituted derivatives shows a monotonic increase with molar mass, while α-substituted derivatives tend to show slightly lower ln(η) than their β-substituted isomers. The effect of branching depends on the chain length: for C3–C4 substituents, iso-alkyl derivatives exhibit slightly higher ln(η) than n-alkyl isomers, whereas for C5–C6 substituents, the trend reverses. For cycloalkyl-substituted derivatives, ln(η) still increases with molar mass but shows significant scatter at similar molar masses, suggesting that the molecular structure also contributes to viscosity variations. The grouped distributions in Figure 6b show that the median ln(η) follows the order fused < spiro < linked, while the ring-type distributions shift upward from Cycle3 to Cnor derivatives. Among all the designed derivatives, α-n-C1 gives the lowest value.

The flash point exhibits a similar mass-dependent trend but with more direct alkyl-structure dependence. As shown in Figure 6c, the flash point increases with molar mass for both alkyl- and cycloalkyl-substituted derivatives. For alkyl-substituted derivatives, n-alkyl isomers generally show higher flash points than iso-alkyl isomers, and β-substituted derivatives tend to show slightly higher flash points than the corresponding α-substituted isomers. The grouped distributions in Figure 6d show trends broadly consistent with those of viscosity. Linked structures and Cnor derivatives are located in the higher flash-point range, whereas fused structures and Cycle3 derivatives are mainly distributed in the lower range. In addition, α-S-Cnor shows the highest flash point among the designed derivatives. Overall, the flash point shows a strong dependence on molecular mass, whereas viscosity is influenced by both molecular mass and structural factors, such as connection topology and the ring type.

Unlike density, M was retained as an explicit descriptor for the viscosity and flash point to account for the mass-dependent baseline contribution. The MLR models used a common descriptor set that included M, the substitution position (I_α_), branching (I_iso_), the ring type (I_Cycle3_, I_Cycle4_, I_Cycle5_, I_Cycle6_, I_Cnor_), and topology (I_Fused_, I_Spiro_), with linked topology used as the reference level for topological descriptors. The descriptor definitions, regression coefficients, model-performance metrics, residual analysis, outlier identification, and leverage-based applicability-domain assessment are summarized in Tables S4.2–S4.5 and Figures S4.1 and S4.2 of the Supplementary Materials.

The finalized regression equations for ln(η) and T_f_ are listed as follows:

$$\ln(\eta )\;=\;-1.460\;+\;0.021M\;-\;0.075I_{\alpha }\;-\;0.025I_{\mathrm{iso}}\;+\;0.577I_{\mathrm{Cycle}3}\;+\;0.518I_{\mathrm{Cycle}4\;}+\;0.607I_{\mathrm{Cycle}5}$$

$$+0.785I_{\mathrm{Cycle}6}+0.878I_{\mathrm{Cnor}\;}-0.299I_{\mathrm{Fused}}-0.154I_{\mathrm{Spiro}}$$

$$T_{f}=206.240+0.863M-2.520I_{\alpha }-8.564I_{\mathrm{iso}}+12.891I_{\mathrm{Cycle}3}+11.828I_{\mathrm{Cycle}4}+14.364I_{\mathrm{Cycle}5}+15.521I_{\mathrm{Cycle}6}$$

$$+17.833I_{\mathrm{Cnor}\;}-8.075I_{\mathrm{Fused}}-5.228I_{\mathrm{Spiro}}$$

**Figure 7:**
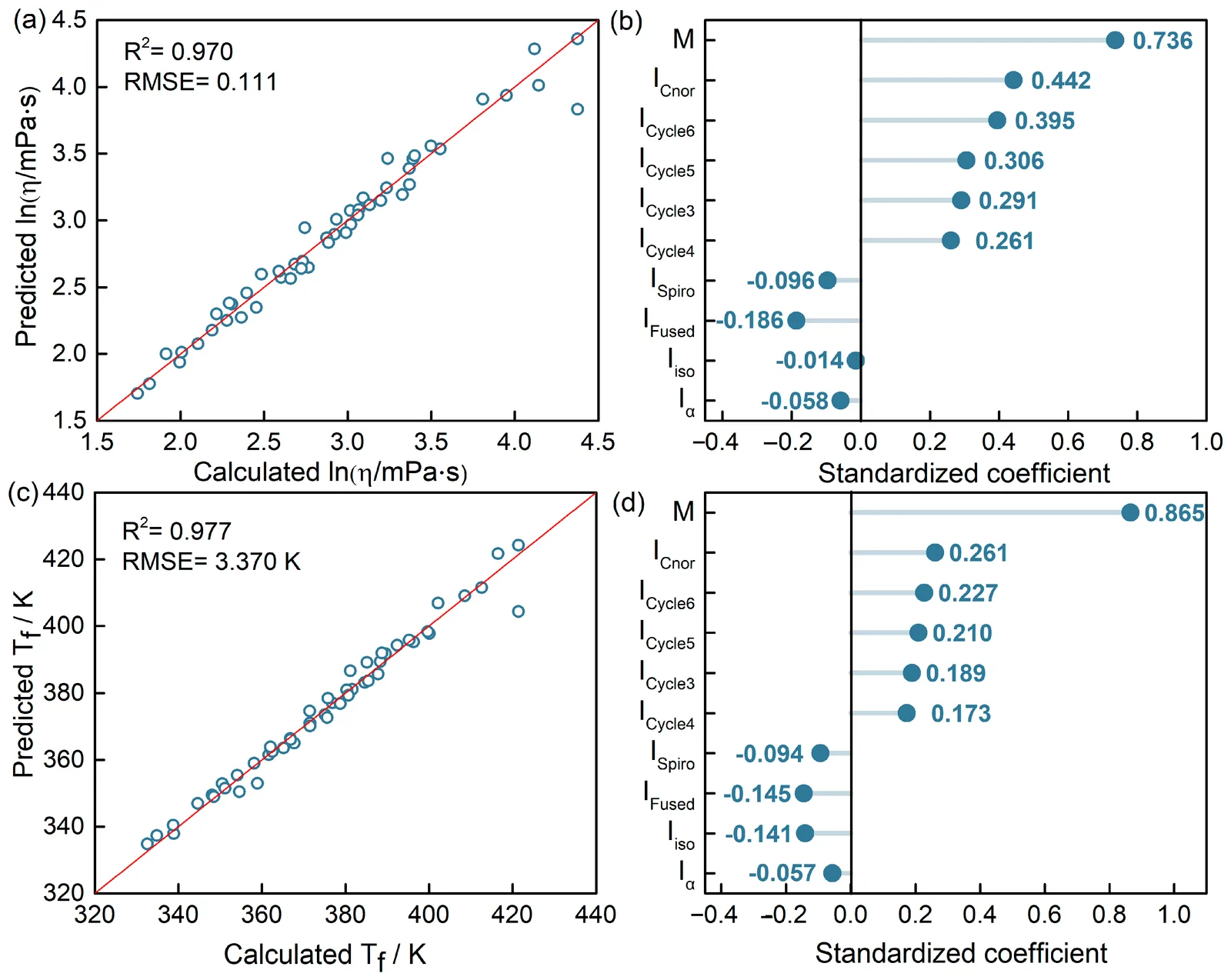
ln(η) and T_f_ model performance and descriptor effects. Predicted versus calculated (**a**) ln(η) and (**c**) T_f_. Descriptor effects on (**b**) ln(η) and (**d**) T_f._

Both models show good statistical performance. The ln(η) model gives an adjusted R^2^ = 0.963, Q^2^ = 0.949, and RMSE = 0.111, while the T_f_ model gives an adjusted R^2^ = 0.971, Q^2^ = 0.960, and RMSE = 3.370 K. As shown in Figure 7, the predicted values agree well with the calculated values for both properties. The standardized coefficient of M is the largest in both models, particularly for $T_{f}$, indicating a strong mass-dependent baseline. After accounting for M, ring-type descriptors remain important, following the same order of coefficient magnitude: I_Cnor_ > I_Cycle6_ > I_Cycle5_ > I_Cycle3_ > I_Cycle4_. This indicates that Cnor and Cycle6 derivatives induce the largest positive shifts in both ln(η) and T_f_. Topology shows a similar estimated tendency in the two models. Relative to linked topology, the negative coefficients of $I_{Fused}$ and $I_{Spiro}$ indicate lower $ln\left(\eta \right)$ and $T_{f}$, giving the order linked > spiro > fused. The main difference between the two models lies in the branching and substitution position. I_iso_ is insignificant in the ln(η) model but has a significant negative contribution to T_f_, indicating that branching lowers the flash point more systematically than viscosity. The I_α_ coefficients are small in magnitude but statistically significant in both models. Residual and applicability-domain analyses are provided in the Supplementary Materials, where potential response outliers and the leverage-defined model domain are discussed.

The viscosity and flash point are influenced by related structural variables but represent distinct physical processes. Viscosity reflects resistance to molecular rearrangement in the liquid phase [30], whereas the flash point is associated with the formation of an ignitable vapor concentration and is therefore closely related to volatility [31]. Molecular mass, geometry, and intermolecular interactions can influence both properties, but through these different mechanisms [32,33].

The weak positional effect can be attributed to the limited structural change caused by α/β substitution. Because the α-site is closer to the decalin core, α-substitution slightly reduces the outward substituent extension and modifies intermolecular contact. However, positional isomerism does not change the chain length, branching mode, or rotatable-bond number; therefore, its influence on intermolecular interactions remains limited, leading to only small changes in the viscosity and flash point. Branching shows no consistent overall effect on viscosity, whereas its influence on the flash point is more systematic. For the flash point, iso-alkyl substitution interrupts continuous chain extension and concentrates the substituent closer to the *trans*-decalin scaffold, which may facilitate molecular escape and thereby lower $T_{f}$. The ring-type effect reflects the combined influence of molecular rigidity, conformational behavior, and effective intermolecular contact. Cnor derivatives exhibit the largest positive contribution to both ln(η) and T_f_, consistent with their rigid bridged skeleton and restricted conformational freedom, which hinder molecular rearrangement in the liquid phase and suppress molecular escape. Cycle6 derivatives also exhibit strong positive contributions, likely because their relatively stable low-energy conformational space and larger ring framework promote more persistent intermolecular contact. Cycle5 derivatives fall as intermediates, whereas Cycle3 and Cycle4 derivatives show weaker positive contributions. This demonstrates that the local geometric constraint alone does not necessarily lead to the strongest increase in the viscosity or flash point. The ring-type contribution is determined by the combined effects of ring rigidity, conformational restriction, and effective intermolecular contact. The topology effect mainly affects the extent of intermolecular contact. Linked topology generally gives the highest values, as the substituent ring projects farther outward from the *trans*-decalin scaffold through an external single-bond linkage, producing a more elongated contour and more extensive intermolecular contact. Fused topology forms a more integrated and less outward-extending contour, reducing effective contact and giving lower viscosity and flash point. Spiro topology remains intermediate, as it is less elongated than linked topology but more three-dimensional than fused topology.

In summary, the viscosity (233.15 K) and flash point were used as auxiliary engineering descriptors for low-temperature fluidity and handling safety, respectively. Both properties generally increase with molecular mass, while molecular geometry provides additional structural modulation. For alkyl-substituted derivatives, branching has no consistent overall effect on viscosity but systematically lowers flash point, possibly by disrupting continuous intermolecular contact and facilitating molecular escape. For cycloalkyl-substituted derivatives, the viscosity and flash point show an estimated topology-related order of linked > spiro > fused, with rigid norbornyl and six-membered-ring derivatives showing the highest values. Fused topology is associated with lower viscosity and flash points, whereas linked topology is associated with higher values, consistent with the differences in molecular contour and intermolecular contact. Therefore, the viscosity and flash point should be considered together as distinct but jointly relevant engineering constraints.

### 2.4. Multi-Objective Screening of HEDF Candidates

**Figure 8:**
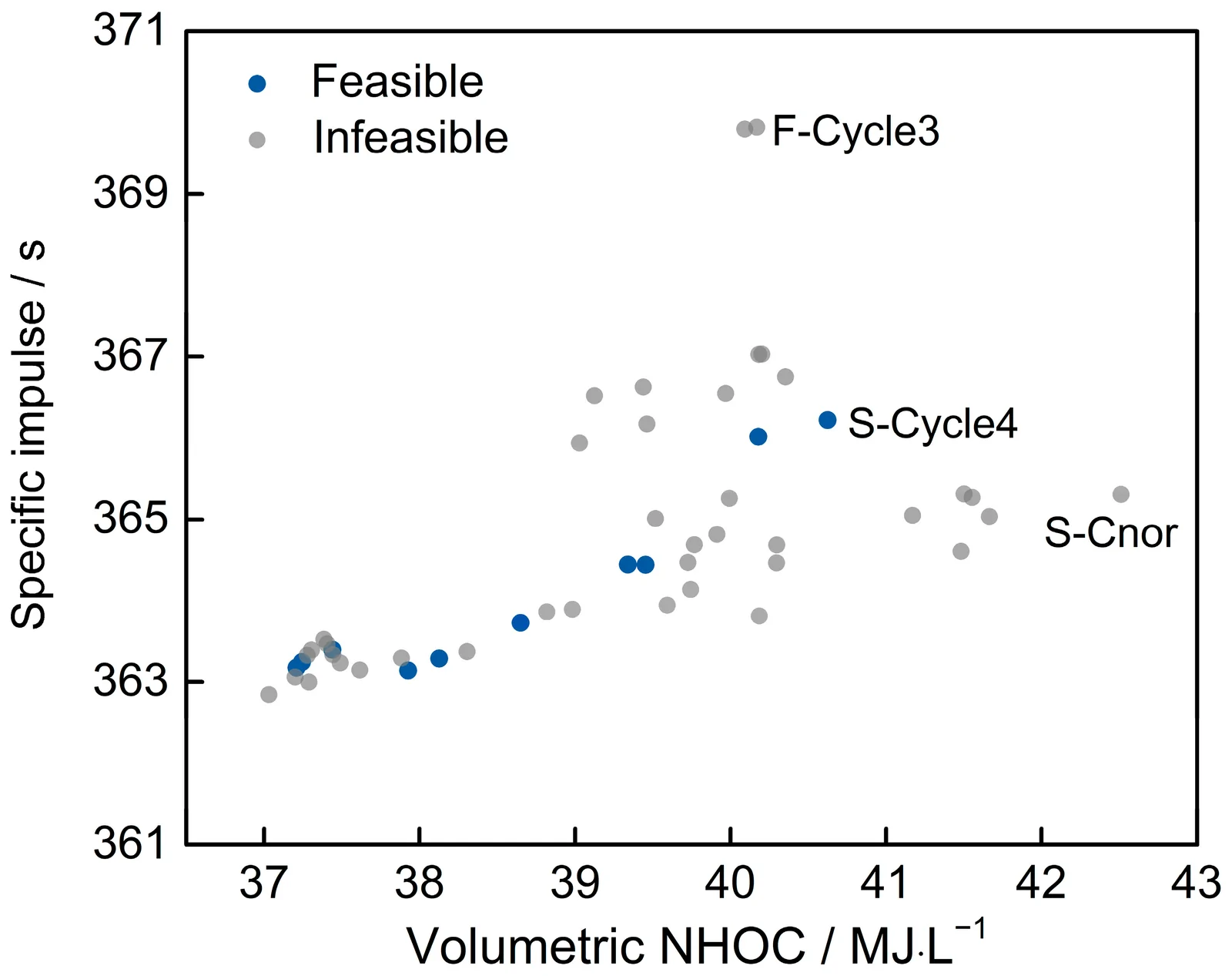
Multi-objective screening in the volumetric NHOC–specific impulse space under the feasibility constraints of η ≤ 16 mPa·s and T_f_ ≥ 360 K. Candidates satisfying both constraints based on the calculated property values are highlighted, and α-S-Cycle4 is identified as the leading candidate within this screening window.

The preceding structure–property analysis reveals distinct trade-offs between the key fuel properties. Because these properties respond differently to structural variation, no single candidate within the present molecular library is expected to simultaneously optimize all performance metrics. A feasibility-constraint-based multi-objective screening strategy was therefore adopted to identify promising candidates under practical performance constraints, as summarized in Figure 8.

The flash point and viscosity at −40 °C were used as feasibility constraints because they are relevant to handling safety and low-temperature fluidity, respectively [32,33]. Density, gravimetric NHOC, volumetric NHOC, and specific impulse were then used to compare the retained candidates. The boundaries of $T_{f}\geq 360\;K$ and η≤16 mPa·s were used as engineering-oriented screening criteria rather than absolute fuel specifications. The $T_{f}\geq 360$ K boundary approximately removes the lowest 25% of flash-point values in the molecular library, excluding the most volatile candidates while retaining a sufficiently broad design space. After this flash-point pre-screening, the η≤16 mPa·s boundary retains approximately the lowest-viscosity 25% of the flash-point-qualified molecules and therefore defines a strict low-viscosity window. Detailed threshold-sensitivity and uncertainty-aware analyses are provided in Section S5 of the Supplementary Materials.

Under the screening windows of η≤16 mPa·s and $T_{f}\geq 360\;K$, ten molecules satisfy both viscosity and flash-point constraints. Among these candidates, α-S-Cycle4 simultaneously exhibits the highest density, gravimetric NHOC, volumetric NHOC, and specific impulse. Therefore, α-S-Cycle4 is identified as the best-balanced candidate within the defined feasible screening window rather than as a universally optimal molecule over the entire molecular library. β-S-Cycle4 also remains a competitive candidate within the same spiro four-membered structural class, indicating that the S-Cycle4 motif provides a favorable balance between energetic performance, propulsion performance, low-temperature fluidity, and flash-point safety.

Prediction uncertainty was further incorporated into the screening by applying a one-RMSE adverse-error criterion using the validation errors reported in Section 3.3. Under this criterion, three candidates—α-n-C4, α-iso-C4, and α-S-Cycle4—remained within both the viscosity and flash-point limits. α-S-Cycle4 retained the highest density, gravimetric NHOC, volumetric NHOC, and specific impulse among these candidates. Thus, accounting for prediction uncertainty reduces the number of robustly retained candidates but does not change the identification of α-S-Cycle4 as the best-balanced candidate. Detailed uncertainty-aware classifications are provided in Section S5.3 of the Supplementary Materials.

In addition to the balanced candidate identified within the screening analysis, several molecules exhibit outstanding performance in individual aspects. β-F-Cycle3 delivers the highest gravimetric NHOC and specific impulse, demonstrating that highly strained fused–cyclopropane frameworks can enhance energy output. However, its flash point is below the 360 K screening boundary. α-S-Cnor shows the highest volumetric NHOC and density, indicating superior packing efficiency, but its very high viscosity excludes it from the low-viscosity feasible window. Iso-C4 derivatives are retained within the feasible window but do not reach the leading energetic-performance region. Their relatively low predicted viscosity and acceptable flash point suggest that they may still be useful as flow-modifying components in fuel formulations. Thus, these molecules may be considered as functional components for selectively improving specific fuel properties rather than as primary standalone candidates.

### 2.5. BDE-Based Analysis of Initial Bond Activation Relevant to Thermal and Oxidative Degradation

**Figure 9:**
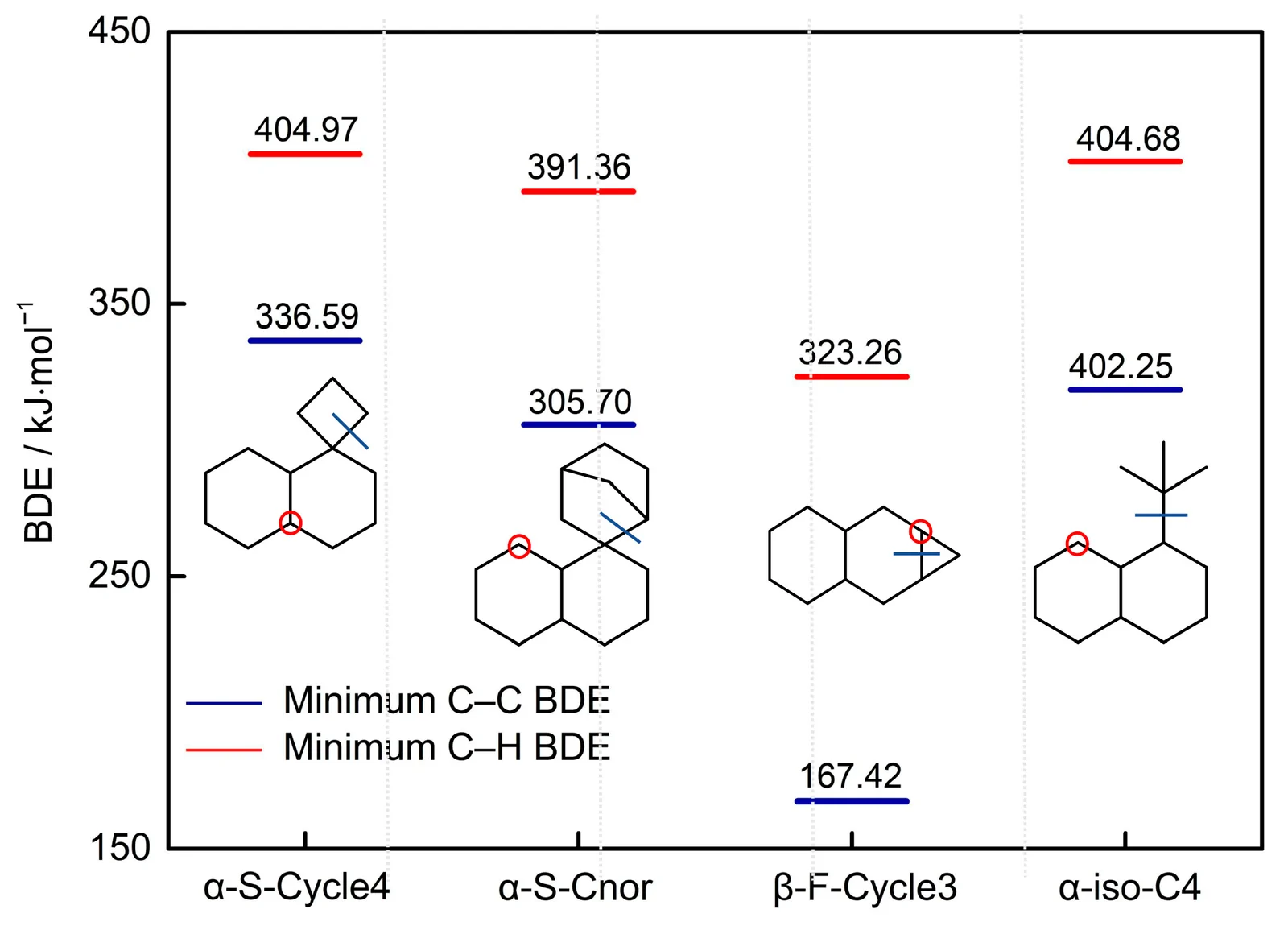
BDE-based analysis of initial bond-activation susceptibility for screening-relevant candidates. Minimum C–C and C–H BDEs are shown in blue and red, respectively. The blue bonds and red circles indicate the weakest C–C bonds and the most labile C–H sites, respectively.

Thermal and oxidative stability are important fuel properties associated with resistance to thermal decomposition and oxidative degradation during storage and operation. To compare the initial bond-activation tendencies of screening-relevant candidates, α-S-Cycle4, β-F-Cycle3, α-S-Cnor, and α-iso-C4 were selected for BDE analysis. These four molecules represent the principal performance priorities identified during screening: α-S-Cycle4 is the best-balanced candidate within the defined feasible window, β-F-Cycle3 exhibits the highest gravimetric NHOC and specific impulse, α-S-Cnor exhibits the highest density and volumetric NHOC, and α-iso-C4 represents a low-viscosity alkyl candidate retained within the feasible window. Thus, the selected set represents the main screening-relevant performance directions rather than a statistically representative subset of the entire molecular library. The minimum C–C and C–H BDE values were used as thermodynamic descriptors of the relative susceptibility to initial C–C homolysis and H abstraction, respectively, as summarized in Figure 9.

For initial C–C homolysis, the minimum BDE values follow the descending order α-S-Cycle4 (370.85 kJ·mol^−1^) > α-S-Cnor (352.48 kJ·mol^−1^) > β-F-Cycle3 (346.83 kJ·mol^−1^) > α-iso-C4 (341.02 kJ·mol^−1^). The comparatively high minimum C–C BDE of α-S-Cycle4 indicates the lowest thermodynamic susceptibility to initial C–C homolysis among the four examined candidates. In contrast, the lower value of β-F-Cycle3 indicates a greater susceptibility to initial C–C bond activation. Therefore, although the fused–cyclopropane motif favors gravimetric energy and propulsion performance, its comparatively weak C–C bond should be considered during candidate evaluation.

For initial H abstraction, the minimum C–H BDE values follow the order α-S-Cycle4 (402.15 kJ·mol^−1^) > β-F-Cycle3 (390.56 kJ·mol^−1^) > α-iso-C4 (387.84 kJ·mol^−1^) > α-S-Cnor (381.67 kJ·mol^−1^). The higher minimum C–H BDE of α-S-Cycle4 indicates lower thermodynamic susceptibility to initial H abstraction within the examined set, whereas the lower value of α-S-Cnor identifies a comparatively more labile C–H site. These results indicate that structural modifications favoring volumetric energy density may introduce trade-offs in initial bond-activation susceptibility.

Overall, the minimum C–C and C–H BDEs provide molecular-level descriptors of the thermodynamic susceptibility to initial C–C homolysis and H abstraction, respectively. They do not account for reaction kinetics, radical chain propagation, oxygen transport, temperature- and pressure-dependent reaction pathways, or other factors governing practical fuel stability. Therefore, the present BDE analysis should not be interpreted as a complete demonstration of thermal or oxidative stability under operating conditions. Within this limited descriptor-based comparison, α-S-Cycle4 exhibits comparatively favorable minimum C–C and C–H BDE values. Combined with its high volumetric NHOC, specific impulse, and acceptable low-temperature fluidity, it remains a promising HEDF candidate, though its practical thermal and oxidative stability requires further kinetic and experimental validation.

### 2.6. Practical Assessment of the Screened Candidates

The multi-objective screening identified α-S-Cycle4 as the candidate with the most balanced overall property profile within the designed molecular library. To place its predicted performance in a broader context, *trans*-decalin was retained as the parent-scaffold reference, while JP-10, 2-ethylnorbornane (EthNB) [34], and exo,exo-tetracyclo[3.3.1.0^2,4^.0^6,8^]nonane (exo-TCN) [35] were selected as representative HEDF benchmarks. JP-10 provides an established high-density fuel reference, whereas EthNB and exo-TCN represent alkyl-substituted norbornane and cyclopropane-strain-based fuel designs, respectively. Specific impulse and stability were not included in Table 1, as reported specific-impulse values depend strongly on propulsion conditions, while the available stability metrics are not directly comparable.

On the common computational basis, α-S-Cycle4 exhibits density, gravimetric NHOC, and volumetric NHOC approximately 9.1%, 2.8%, and 12.1% higher than those of *trans*-decalin, respectively, together with a 35.56 K higher predicted flash point. A direct comparison of viscosity at 233.15 K was not made for *trans*-decalin because of the difference in low-temperature phase conditions. Relative to JP-10, α-S-Cycle4 shows approximately 0.6%, 2.4%, and 3.0% higher calculated density, gravimetric NHOC, and volumetric NHOC, respectively. Its calculated viscosity at 233.15 K is approximately 18.3% lower, while its predicted flash point is 36.20 K higher.

EthNB and exo-TCN illustrate different trade-offs between the fuel properties. EthNB shows the lowest calculated viscosity among the compared fuels, but its lower density and gravimetric NHOC result in a volumetric NHOC of 35.727 MJ·L^−1^, together with a calculated flash point of 306.51 K. Exo-TCN gives the highest calculated density (0.981 g·cm^−3^) and a volumetric NHOC of 40.466 MJ·L^−1^, close to that of α-S-Cycle4 (40.555 MJ·L^−1^). In contrast, α-S-Cycle4 has a higher calculated gravimetric NHOC (43.032 versus 41.25 MJ·kg^−1^) and a slightly higher predicted flash point (362.04 versus 359.67 K) than exo-TCN. Thus, α-S-Cycle4 does not maximize each property individually, but provides a balanced combination of gravimetric and volumetric energy content, low-temperature viscosity, and flash point.

Coal-tar-derived naphthalene provides a potential non-petroleum precursor to the decalin scaffold, although the final substituted candidates are not direct molecular constituents of coal-tar fractions. A plausible retrosynthetic route to α-S-Cycle4 begins with complete hydrogenation of naphthalene to decalin, followed by functionalization of the decalin framework to produce a carbonyl intermediate. Olefination of this intermediate could generate a decalin-derived exocyclic alkene, followed by a cyclobutane-forming [2+2] cycloaddition to construct the spiro–cyclobutane motif. Related functionalization and ring-forming transformations could provide access to the fused, spirocyclic, and bridged structures considered in the molecular library.

The proposed routes represent conceptual retrosynthetic possibilities, and their practical implementation will depend on regioselectivity, stereochemical control, reaction yield, purification requirements, and process economics. Moreover, viscosity at 233.15 K characterizes only one aspect of low-temperature fluidity and does not directly describe melting point, freezing point, pour point, crystallization behavior, or long-term phase stability. The flash point and minimum BDE values similarly provide comparative molecular descriptors rather than complete evaluations of handling safety or thermal and oxidative stability. Experimental synthesis, condition-controlled property measurements, low-temperature phase-behavior analysis, combustion characterization, stability testing, and material-compatibility evaluation are therefore required to establish the practical fuel performance of the screened candidates.

## 3. Computational Methods

### 3.1. Molecular Library Design

Decalin was selected as the parent scaffold for constructing the molecular library. Global conformational sampling showed that both *cis*- and *trans*-decalin are dominated by a single low-energy conformer at 298.15 K, with all other conformers lying more than 20.9 kJ mol^−1^ higher in energy. The two stereoisomers exhibit distinct ring-fusion geometries. *trans*-decalin adopts an e,e ring-fusion geometry with two chair cyclohexane rings, resulting in a relatively extended conformation, whereas *cis*-decalin adopts an e,a ring-fusion geometry with a folded backbone and local steric crowding. The Gibbs free energy of *cis*-decalin is 11.9 kJ mol^−1^ higher than that of *trans*-decalin, indicating that the *trans* isomer is thermodynamically favored. Therefore, all derivatives in this work were constructed on the *trans*-decalin scaffold.

**Figure 10:**
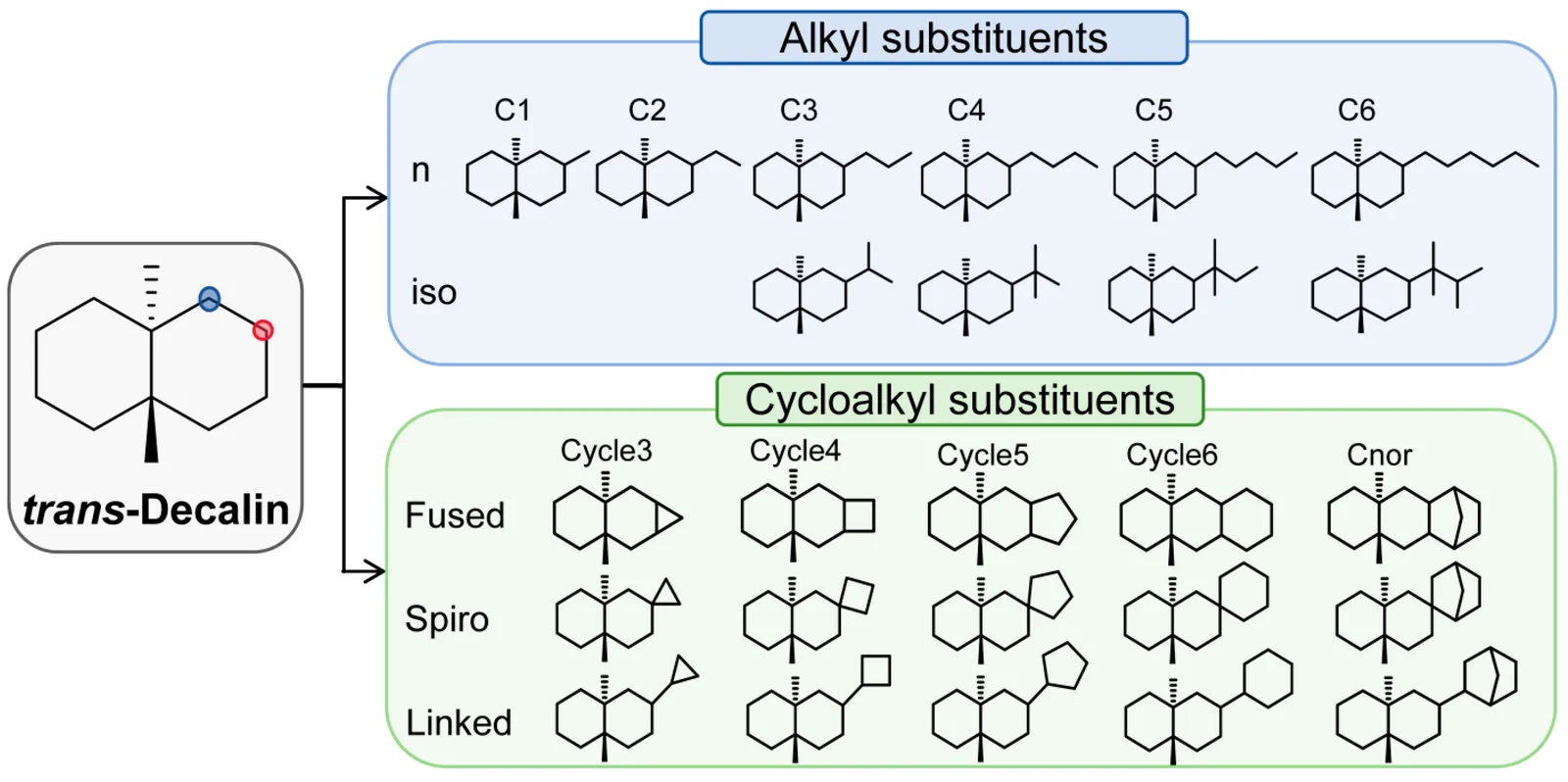
Molecular library design for substituted *trans*-decalin derivatives.

A virtual library of *trans*-decalin derivatives was constructed to investigate structure–property relationships of decalin-based HEDF candidates, as shown in Figure 10. Substituents were introduced at two non-equivalent positions of the *trans*-decalin scaffold, denoted as the α- and β-sites. Two classes of substituents were considered. The first class included linear and branched alkyl groups containing one to six carbon atoms, while the second consisted of cycloalkyl substituents with different ring sizes and connection topologies, including fused, spiro, and linked structures. Norbornyl-type substituents were also included as bridged cyclic substituents. The structural notation used for the molecular library is provided in the Supplementary Materials.

The *trans*-decalin scaffold was substituted at two non-equivalent positions, denoted as α- and β-sites. Two substituent classes were considered: (1) linear/branched alkyl groups (C1–C6); and (2) cycloalkyl substituents with varying ring sizes (Cycle3–Cycle6), connection topologies (fused/F, spiro/S, linked/L), and bridged norbornyl (Cnor) groups. The molecular library covered key structural variations to systematically investigate structure–property relationships.

### 3.2. Computational Procedures and Property Evaluation

Global conformational sampling was performed for all designed molecules using CREST v2.12.2 at the GFN2-xTB level [36,37]. Conformers within 12.5 kJ mol^−1^ of the lowest-energy conformer were optimized and subjected to harmonic frequency calculations at the M06-2X/6-31+G(d,p) level, followed by single-point energy calculations at the M06-2X/def2-TZVP level using Gaussian 16 [38,39]. The M06-2X/def2-TZVP single-point energies were combined with the thermal corrections obtained from the frequency calculations to determine the relative Gibbs free energies at 298.15 K, which were used for Boltzmann averaging of conformer-dependent properties [40]. Further details are provided in Section S1.3 of the Supplementary Materials.

Liquid densities were calculated using GROMACS 2024.5 with the OPLS-AA force field [41,42]. Molecular topologies and force-field parameters were generated using LigParGen [43]. Each system contained 500 identical fuel molecules in a molecule-specific cubic box with three-dimensional periodic boundary conditions. Following energy minimization, the systems were equilibrated for 500 ps in the NVT ensemble at 298.15 K, followed by 1 ns in the NPT ensemble at 298.15 K and 1 bar. A 10 ns NPT production simulation was subsequently performed using a 2 fs time step, and the final 5 ns was used for density analysis. Detailed simulation settings, convergence assessment, and statistical uncertainty estimation are provided in Section S1.4 of the Supplementary Materials. COSMO-RS calculations, implemented in COSMOthermX 2022, and the Politzer empirical correlation were also used for comparison [44,45,46], whereas all subsequent structure–property analyses were based on the MD-derived densities.

Net heat of combustion (NHOC) at 298.15 K was determined from DFT-calculated standard enthalpies of combustion. Gas-phase standard enthalpies of formation were obtained by applying Hess’s law to compound-specific isodesmic reactions [47], and the corresponding liquid-phase values were derived using COSMO-RS-calculated enthalpies of vaporization. Complete combustion to CO_2_(g) and H_2_O(g) was assumed. NHOC values were reported as positive magnitudes of the combustion-energy release. The isodesmic reaction schemes, reference data, and detailed equations are provided in Section S1.5 of the Supplementary Materials. Specific impulse ($I_{sp}$) was calculated using Rocket Propulsion Analysis (RPA) v.2.3 [48], with liquid oxygen, O_2_(l), as the oxidizer. The oxidizer coefficient, combustion-chamber pressure, and chamber-to-exit pressure ratio were fixed at 0.7, 7 MPa, and 70, respectively, for all fuel candidates. The reported values correspond to the theoretical vacuum specific impulse. Detailed thermochemical inputs and calculation settings are provided in Section S1.6 of the Supplementary Materials. Viscosity (η) and flash points (T_f_) were predicted using COSMO-RS, as implemented in COSMOthermX 2022, with the BP-TZVP-FINE parameterization and the conformer ensemble described above. Dynamic viscosity was evaluated at 233.15 K (−40 °C) as a descriptor of low-temperature fluidity [49]. Detailed calculation settings are provided in Section S1.7 of the Supplementary Materials.

Multiple linear regression (MLR) models were constructed using physically interpretable structural descriptors to quantify structure–property relationships and were fitted by ordinary least squares. Model performance was evaluated using the coefficient of determination (R^2^), adjusted R^2^, cross-validated Q^2^, root-mean-square error (RMSE), and mean absolute error (MAE), while multicollinearity was assessed using variance inflation factors (VIFs). Residual analysis, outlier identification, and leverage-based applicability-domain assessment were performed for all regression models to evaluate predictive reliability and avoid extrapolation beyond the investigated structural space [50]. In addition, selected microscopic descriptors were calculated to support the mechanistic interpretation. The regression methodology, internal-validation procedures, and leverage-based applicability-domain assessment are described in Section S1.8 of the Supplementary Materials.

For the representative candidates included in the stability analysis, homolytic C–C and C–H bond dissociation enthalpies (BDEs) were calculated to characterize susceptibility to initial C–C bond homolysis and H abstraction, respectively. Homolytic BDEs provide a thermodynamic measure of the energy required for initial homolytic bond cleavage in organic molecules [51]. The minimum C–C BDE was used as a comparative descriptor of susceptibility to initial C–C bond homolysis, whereas the minimum C–H BDE was used as a comparative descriptor of susceptibility to initial H abstraction [52]. These descriptors characterize only the thermodynamics of the initial bond-breaking steps and do not constitute complete predictions of thermal or oxidative fuel stability under practical operating conditions. Detailed computational procedures are provided in Section S1.9 of the Supplementary Materials.

### 3.3. Validation of the Computational Workflow

The prediction protocols for the liquid density, gravimetric net heat of combustion (NHOC), dynamic viscosity, and flash point were quantitatively evaluated against experimental data for representative hydrocarbons. To ensure comparability, the benchmark data were evaluated using consistent property definitions, physical states, units, and temperatures where applicable. Density was compared at 298.15 K, gravimetric NHOC was expressed on the same liquid-fuel net-heat basis for complete combustion to CO_2_(g) and H_2_O(g), and dynamic viscosity was compared at the corresponding experimental temperature. Because viscosity at 233.15 K was used in the subsequent screening, the validation data at this temperature were also evaluated separately.

For liquid density (*n* = 10), the MAE and RMSE were 0.003 and 0.003 g·cm^−3^, respectively. For gravimetric NHOC (*n* = 10), the corresponding values were 0.500 and 0.542 MJ·kg^−1^. The viscosity dataset contained 13 condition-matched points, giving an MAE of 1.833 mPa·s and an RMSE of 2.107 mPa·s. For the 233.15 K subset (*n* = 10), the MAE and RMSE were 1.964 and 2.267 mPa·s, respectively. Flash-point validation (*n* = 10) gave an MAE of 1.773 K and an RMSE of 1.853 K. The complete benchmark data, additional statistical metrics, and experimental–calculated parity plots are provided in Section S1.10 of the Supplementary Materials.

Volumetric NHOC was not independently validated because it was derived from liquid density and gravimetric NHOC. Specific impulse was not included in the direct statistical validation because it depends on the oxidizer and propulsion conditions; identical RPA settings were therefore applied to all candidate fuels for internally consistent comparison.

## 4. Conclusions

Density and volumetric energy density are governed by molecular mass and packing efficiency, enhanced by alkyl branching, α-substitution, spiro topology, and compact cycloalkyl rings. Gravimetric energy and specific impulse depend primarily on the H/C ratio and ring strain, with strained small rings boosting propulsion performance. The flash point is mainly associated with molecular mass, whereas viscosity is affected by both molecular mass and structural factors, such as connection topology and ring type. Fused topology improves fluidity, while linked topology enhances safety; short branched alkyl groups act as flow modifiers.

Multi-objective screening identifies the spiro four-membered ring (S-Cycle4) as the optimal balanced scaffold, combining high volumetric energy, good propulsion performance, and acceptable viscosity and flash points with favorable BDE characteristics associated with lower susceptibility to thermal- and oxidative-degradation-related initial bond activation. β-F-Cycle3, α-S-Cnor, and α-iso-C4 may serve as functional blending components for targeted performance enhancement.

This work establishes quantitative structure-based guidelines for the rational design of decalin-derived HEDF candidates. These findings provide quantitative guidance for the rational design of decalin-derived HEDFs and highlight abundant, industrially separable coal-tar-derived naphthalene as a promising non-petroleum precursor to the decalin scaffold. The practical viability of the proposed candidates remains to be experimentally established.

## Figures and Tables

**Table 1:** Comparison of the fuel-relevant properties of α-S-Cycle4 with *trans*-decalin and JP-10.

Fuel	Density/g·cm^−3^ (T/K)	Gravimetric NHOC/MJ·kg^−1^	Volumetric NHOC/MJ·L^−1^	Viscosity (T/K)	Flash Point/K
**Reported experimental data**					
*trans*-Decalin	0.866 (298.15)	42.54	36.84	1.956 mPa·s (298.15)	326.85
JP-10	0.932 (298.15)	42.18	39.312	17.70 mPa·s (233.15)	327.55
EthNB	0.860 (298.15)	42.8	36.808	5.47 mPa·s (233.15)	313.02
exo-TCN	0.990 (298.15)	42.7	42.273	13.1 mm^2^·s^−1^ (233.15) *	—
**Calculated data—this work**					
*trans*-Decalin	0.864 (298.15)	41.88	36.184	— †	326.48
JP-10	0.937 (298.15)	42.04	39.391	16.314 mPa·s (233.15)	325.84
EthNB	0.858 (298.15)	41.64	35.727	4.620 mPa·s (233.15)	306.51
exo-TCN	0.981 (298.15)	41.25	40.466	11.950 mPa·s (233.15)	359.67
α-S-Cycle4	0.9424 (298.15)	43.032	40.555	13.326 mPa·s (233.15)	362.04

Notes: Experimental values are listed under their reported conditions. Volumetric NHOC was calculated from the corresponding density and gravimetric NHOC. * Kinematic viscosity: the calculated values are dynamic viscosities. † No 233.15 K liquid-viscosity value was used for *trans*-decalin because of the low-temperature phase condition.

## Data Availability

The original contributions presented in this study are included in the article/Supplementary Materials. Further inquiries can be directed to the corresponding author.

## Supplementary Materials

The following supporting information can be downloaded at: https://www.mdpi.com/article/10.3390/molecules31162911/s1, Refs. [53,54,55,56,57,58,59,60,61,62,63,64,65,66,67,68,69,70,71,72,73] are cited in Supplementary Materials.

## Nomenclature

Abbreviation	Full Name	Unit
BDE	Bond dissociation enthalpy	kJ·mol^−1^
C1–C6	Alkyl substituents containing one to six carbon atoms	/
Cnor	Norbornyl-type substituent	/
Cycle3	Three-membered cycloalkyl substituent	/
Cycle4	Four-membered cycloalkyl substituent	/
Cycle5	Five-membered cycloalkyl substituent	/
Cycle6	Six-membered cycloalkyl substituent	/
E_strain_	Ring-strain energy	kJ·mol^−1^
F	Fused connection topology	/
Gravimetric NHOC	Net heat of combustion per unit mass	MJ·kg^−1^
H/C	Atomic hydrogen-to-carbon ratio	/
HEDF	High-energy-density fuel	/
I_Cnor_	Binary descriptor for norbornyl-type substituent	/
I_Cycle3_	Binary descriptor for three-membered cycloalkyl substituent	/
I_Cycle4_	Binary descriptor for four-membered cycloalkyl substituent	/
I_Cycle5_	Binary descriptor for five-membered cycloalkyl substituent	/
I_Cycle6_	Binary descriptor for six-membered cycloalkyl substituent	/
I_Fused_	Binary descriptor for fused connection topology	/
I_Linked_	Binary descriptor for linked connection topology	/
I_Spiro_	Binary descriptor for spiro connection topology	/
I_iso_	Binary descriptor for iso-alkyl branching	/
I_α_	Binary descriptor for α-substitution	/
I_sp_	Specific impulse	s
L	Linked connection topology	/
ln(N_conf_+1)	Natural logarithm of the number of accessible conformers plus one	/
ln(η)	Natural logarithm of dynamic viscosity	/
LOX	Liquid oxygen	/
M	Molar mass	g·mol^−1^
MD	Molecular dynamics	/
MLR	Multiple linear regression	/
N	Number of particles	
N_C_	Alkyl carbon number/alkyl chain length descriptor	/
NPT	Isothermal–isobaric ensemble with constants N, P, and T	/
P	Pressure	MPa
*p*	Statistical *p*-value	/
Q^2^	Cross-validated coefficient of determination	/
R^2^	Coefficient of determination	/
R_g_	Radius of gyration	Å
RMSE	Root-mean-square error	/
S	Spiro connection topology	/
T	Temperature	K
T_f_	Flash point	K
V_free_	Intermolecular free volume	cm^3^·mol^−1^
V_free_/M	Mass-normalized intermolecular free volume	cm^3^·g^−1^
V_int_	Intrinsic occupied molecular volume	cm^3^·mol^−1^
V_int_/M	Mass-normalized intrinsic occupied volume	cm^3^·g^−1^
V_m_	Molar volume	cm^3^·mol^−1^
V_m_/M	Mass-normalized molar volume; reciprocal of density	cm^3^·g^−1^
Volumetric NHOC	Net heat of combustion per unit volume	MJ·L^−1^
η	Dynamic viscosity	mPa·s
ρ	Liquid density	g·cm^−3^
σ(V)	Standard deviation of conformer volume/conformational volume dispersion	cm^3^·mol^−1^
χ_asph_	Molecular asphericity descriptor	/
