# Supplementary material for: Rational Design and Structure–Activity Relationship Analysis of Decalin-Based High-Energy-Density Fuel Molecules
Source: Molecules. 2026 Aug 20;31(16):2911. doi: 10.3390/molecules31162911 (PMC13515900; doi:10.3390/molecules31162911)
Supplement: Supplementary file 1 [file molecules-31-02911-s001.zip › Highlights_Molecules.pdf]

Highlights:

- 52 decalin-based fuel molecules from coal naphthalene are systematically evaluated.
- Ring structure and connection type dominate fuel property variations.
- S-Cycle4 achieves optimal balance between energy density and operability.
- Three blending components are identified for customized fuel formulations.
