# Supplementary material for: Rational Design and Structure–Activity Relationship Analysis of Decalin-Based High-Energy-Density Fuel Molecules
Source: Molecules. 2026 Aug 20;31(16):2911. doi: 10.3390/molecules31162911 (PMC13515900; doi:10.3390/molecules31162911)
Supplement: Supplementary file 1 [file molecules-31-02911-s001.zip › Supporting Information0816.pdf]

Supporting Information

S1. Computational methods

S1.1 Fundamental framework

Decalin was used as the parent molecular scaffold. Both cis- and trans-decalin stereoisomers were examined, and their relative Gibbs free energies at 298.15 K are summarized in Table S1.1. Based on these results, the trans isomer was selected as the scaffold for all molecules in this work.

Table S1.1 Relative Gibbs free energies of decalin stereoisomers at 298.15 K.

Isomer	ΔG / kJ/mol
trans	0
cis	11.90

S1.2 Molecular notation

In the notation adopted here, substitution positions are denoted as α and β. Linear alkyl substituents are denoted as n-Cn, while branched alkyl substituents are denoted as iso-Cn, where n represents the number of carbon atoms in the substituent. Cycloalkyl substituents are denoted as Cycle3-Cycle6, corresponding to three- to six-membered rings. Norbornyl-type substituents featuring a bridged-ring structure are denoted as Cnor. The connection mode between the cycloalkyl substituent and the decalin scaffold is classified as fused (F), spiro (S), or linked (L).

Table S1.2 Definition of structural notation used for decalin derivatives.

Notation	Description
α / β	Substitution site on the decalin scaffold
n-Cn	Linear alkyl chain containing n carbon atoms
iso-Cn	Branched alkyl chain containing n carbon atoms
Cycle3–Cycle6	Three- to six-membered cycloalkyl substituents
Cnor	Norbornyl-type substituent
F	Fused topology
S	Spiro topology

S1.3 Conformational sampling and Boltzmann averaging

Conformational sampling was performed in the gas phase using CREST 2.12.2 at the GFN2-xTB level. For each molecule, conformers within 3.0 kcal·mol^{-1} of the lowest-energy conformer were retained for subsequent DFT calculations.

Geometry optimizations and harmonic frequency calculations were performed at the M06-2X/6-31+G(d,p) level using Gaussian 16. All retained conformers were confirmed as local minima by the absence of imaginary frequencies. Single-point electronic energies were subsequently calculated at the M06-2X/def2-TZVP level. The Gibbs free energy used for conformer weighting was obtained by combining the M06-2X/def2-TZVP single-point electronic energy with the thermal correction derived from the M06-2X/6-31+G(d,p) frequency calculation:

$$G_i = E_i^{\mathrm{SP}} + \left(G_i^{\mathrm{freq}} - E_i^{\mathrm{freq}}\right) \tag{S1}$$

where E_i^{SP} is the M06-2X/def2-TZVP single-point electronic energy, and $G_i^{\mathrm{freq}}-E_i^{\mathrm{freq}}$ is the Gibbs free-energy thermal correction obtained at the M06-2X/6-31+G(d,p) level.

The Boltzmann weight of conformer i at 298.15 K was calculated as:

$$w_i = \frac{\exp\left(-\Delta G_i/RT\right)}{\sum_{j=1}^{n}\exp\left(-\Delta G_j/RT\right)} \tag{S2}$$

where $\Delta G_i = G_i - G_{\mathrm{min}}$, R is the gas constant, and T = 298.15 K. All conformer-dependent properties were obtained by Boltzmann averaging:

$$\bar{P} = \sum_{i=1}^{n} w_i P_i \tag{S3}$$

where P_i is the property calculated for conformer i.

S1.4 Liquid density (ρ)

Politzer method: The ρ was calculated using the Politzer empirical correlation:

$$\rho = \alpha\left(\frac{M}{V(0.001)}\right) + \beta v(\sigma_{tot}^2) + \gamma \tag{S4}$$

where M is the molecular molar mass, $V_{0.001}$ is the molecular volume corresponding to the 0.001 electrons·bohr^{-3} electronic-density isosurface, and $v(\sigma_{tot}^2)$ is molecular surface electrostatic interaction descriptor. The coefficients were $\alpha = 0.9183$, $\beta = 0.0028$, $\gamma = 0.0443$. The molecular descriptors ($V_{0.001}$, $v(\sigma_{tot}^2)$) were computed using Multiwfn 3.8 software. The conformer-specific density values were Boltzmann-averaged using the conformer weights described in Section S1.3.

COSMO-RS method: Liquid density was also predicted at 298.15 K using COSMO-RS as implemented in COSMOthermX 2022, with the BP-TZVP-FINE parameterization. Corresponding COSMO input files were generated from Gaussian 16 calculations performed at the BP86/def2-TZVP level. The conformer-specific density

values were Boltzmann-averaged using the conformer weights described in Section S1.3.

MD simulation: Molecular dynamics simulations were performed using GROMACS 2024.5 with the OPLS-AA force field. Molecular topologies and force-field parameters were generated using LigParGen. Each simulation system contained 500 identical fuel molecules in a cubic box with three-dimensional periodic boundary conditions. Because the investigated molecules differed in molecular size, the initial box dimensions were determined separately for each system. The initial cubic box-edge length used for every system is reported in Supporting Data. Following energy minimization, the systems were equilibrated for 500 ps in the NVT ensemble at 298.15 K using the V-rescale thermostat, followed by 1 ns of NPT equilibration at 298.15 K and 1 bar using the C-rescale barostat. A total of 10 ns of NPT production simulation was subsequently performed at 298.15 K and 1 bar using the V-rescale thermostat and the Parrinello–Rahman barostat. A time step of 2 fs was used throughout the equilibration and production simulations. Liquid density was calculated by averaging the final 5 ns of the production trajectory. Density convergence was assessed from the density time series and the corresponding running average over the final 5 ns. The absence of systematic drift in the density time series and stabilization of the running average were used as the convergence criteria. Statistical uncertainty was estimated by dividing the final 5 ns into five consecutive 1 ns block. The mean density was calculated for each block, and the standard error of the five block-averaged densities was used as the uncertainty estimate. The resulting density values and statistical uncertainties are reported in Supporting Data.

Among the three methods, MD-derived densities were used for subsequent structure-property analyses in the main text.

S1.5 Net Heat of Combustion (NHOC)

The NHOC values were determined from standard reaction enthalpies at 298.15 K. Gas-phase standard enthalpies of formation, $\Delta_f H^{\ominus}_{\text{gas}}$, were obtained by applying Hess's law to isodesmic reactions that conserve the numbers and types of chemical bonds between the reactants and products. Structurally analogous reference compounds were used: linear and branched alkanes for alkyl-substituted systems, and cyclohexane and methylcyclohexane for cyclic-substituted systems. The specific isodesmic reaction schemes, reference compounds, and experimental gas-phase formation enthalpies are listed in Supporting Data.

The standard enthalpy of each species at 298.15 K was obtained by combining the M06-2X/def2-TZVP single-point electronic energy with the thermal enthalpy correction derived from the M06-2X/6-31+G(d,p) frequency calculation:

$$H_i^{\ominus} = E_i^{\text{SP}} + \left(H_i^{\text{freq}} - E_i^{\text{freq}}\right) \tag{S5}$$

where E_i^{SP} is the M06-2X/def2-TZVP single-point electronic energy, and $H_i^{\text{freq}}-E_i^{\text{freq}}$ is the thermal enthalpy correction obtained at the M06-2X/6-31+G(d,p) level.

For a general isodesmic reaction, the standard reaction enthalpy was calculated as:

$$\Delta_r H^\ominus_{\text{iso}} = \sum_j v_j H^\ominus_j(\text{products}) - \sum_i v_i H^\ominus_i(\text{reactants}) \tag{S6}$$

The gas-phase standard enthalpy of formation of the target compound was then obtained by rearranging the corresponding Hess-law expression using the experimental formation enthalpies of the reference compounds.

The liquid-phase standard enthalpy of formation was obtained as:

$$\Delta_f H^\ominus_{\text{liq}} = \Delta_f H^\ominus_{\text{gas}} - \Delta_{\text{vap}} H^\ominus \tag{S7}$$

where $\Delta_{\text{vap}}H^\circ$ was obtained from COSMO-RS calculations.

For a hydrocarbon fuel with the molecular formula C_xH_y, the complete combustion reaction used to calculate NHOC was:

$$C_xH_y(l) + \left(x + \frac{y}{4}\right) O_2(g) \rightarrow xCO_2(g) + \frac{y}{2} H_2O(g) \tag{S8}$$

The standard enthalpy of combustion was calculated as:

$$\Delta_c H^\ominus = x\Delta_f H^\ominus[CO_2(g)] + \frac{y}{2}\Delta_f H^\ominus[H_2O(g)] - \Delta_f H^\ominus[C_xH_y(l)] - \left(x + \frac{y}{4}\right)\Delta_f H^\ominus[O_2(g)] \tag{S9}$$

where $\Delta_f H^\ominus[O_2(g)] = 0$. Gravimetric NHOC was reported as a positive quantity:

$$\text{Gravimetric NHOC} = \frac{|\Delta_c H^\ominus|}{M} \tag{S10}$$

Volumetric NHOC was calculated as:

$$\text{Volumetric NHOC} = \rho \cdot \text{NHOC}_m \tag{S11}$$

where M is the molar mass and ρ is the MD-derived liquid density.

S1.6 Specific impulse (I_{sp})

The theoretical vacuum specific impulse of each fuel was calculated using Rocket Propulsion Analysis (RPA) v.2.3 with liquid oxygen, $O_2(l)$, as the oxidizer. Each fuel was specified by its molecular formula, liquid phase, and standard enthalpy of formation at 298.15 K. The liquid-phase standard enthalpies of formation obtained using the isodesmic-reaction and COSMO-RS procedures described in Section S1.5 were used as the thermochemical inputs.

For all fuel candidates, the oxidizer excess coefficient, α_{ox}, was fixed at 0.7. The coefficient was defined as the ratio of the actual oxidizer-to-fuel mass ratio to the stoichiometric oxidizer-to-fuel mass ratio:

$$\alpha_{ox} = \frac{(O/F)_{\text{actual}}}{(O/F)_{\text{stoich}}} \tag{S12}$$

Thus, $\alpha_{ox} = 0.7$ corresponds to a fuel-rich condition in which the actual oxidizer-to-fuel mass ratio is 70% of the stoichiometric value.

The combustion-chamber pressure was set to 7 MPa, and the chamber-to-exit pressure ratio was fixed at:

$$\frac{P_c}{P_e} = 70 \tag{S13}$$

where P_c and P_e are the combustion-chamber and nozzle-exit pressures, respectively. Chemical equilibrium was assumed in the combustion chamber, and shifting equilibrium was assumed throughout nozzle expansion. The reported I_{sp} values correspond to the theoretical vacuum specific impulse. Identical operating conditions and thermodynamic settings were applied to all fuel candidates to ensure internally consistent comparisons. Therefore, the calculated values were used for comparative molecular screening rather than as predictions of delivered engine performance.

S1.7 Dynamic viscosity (η) and flash point(T_f)

Dynamic viscosity and flash point were predicted using COSMO-RS as implemented in COSMOthermX 2022 with the BP-TZVP-FINE parameterization. The COSMO input files for the retained conformers were generated from Gaussian 16 calculations at the BP86/def2-TZVP level. The same conformer ensemble and Boltzmann weights described in Section S1.3 were used for both properties. Dynamic viscosity was evaluated at 233.15 K (-40 °C) and reported in mPa·s. Flash point was predicted using the flash-point model implemented in COSMOthermX and reported in K. For each molecule, the conformer-dependent property values were Boltzmann-averaged according to Eq. S3 using the conformer weights defined in Eq. S2.

S1.8 Regression analysis

Multiple linear regression (MLR) models were constructed to quantify the contributions of molecular structure to fuel properties and were fitted by ordinary least squares. The general form of the regression model was:

$$Y=\beta_0+\sum_{j=1}^{p}\beta_j X_j+\varepsilon \tag{S14}$$

where Y is the dependent property, X_j is the structural descriptor, β_0 is the intercept, β_j is the regression coefficient, p is the number of descriptors, and ε is the residual term. Different descriptor sets were used for different properties. The descriptor definitions, regression equations, coefficient tables, multicollinearity diagnostics, and model-performance metrics are provided in the corresponding property-specific sections.

Model fitting performance was evaluated using R^2, adjusted R^2, root-mean-square error (RMSE), and mean absolute error (MAE). Internal predictive capability was assessed by leave-one-out cross-validation. The cross-validated coefficient was calculated as:

$$Q^2 = 1-\frac{\sum_{i=1}^{n}(y_i-\hat{y}_{i,-i})^2}{\sum_{i=1}^{n}(y_i-\bar{y})^2} \tag{S15}$$

where $\hat{y}_{i,-i}$ is the predicted value for molecule i obtained from a model fitted after excluding that molecule, and $\bar{y}$ is the mean observed value. Multicollinearity was assessed using variance inflation factors (VIFs). Standardized regression coefficients were used to compare the relative importance of descriptors.

Residual-versus-fitted plots and internally studentized residuals were examined to identify systematic model

deviations and potential response outliers. The internally studentized residual for molecule i was calculated as:

$$r_i = \frac{e_i}{s\sqrt{1\text{-h}_i}} \tag{S16}$$

where $e_i = y_i - \hat{y}_i$ is the residual for molecule i, s is the residual standard error, and h_i is the leverage of molecule i. Molecules with $|r_i| > 3$ were flagged as potential response outliers for further examination but were not automatically excluded from model fitting.

The leverage value was calculated as:

$$\text{h}_i = \mathbf{x}_i^T(\mathbf{X}^T\mathbf{X})^{-1}\mathbf{x}_i \tag{S17}$$

where $\mathbf{X}$ is the model matrix and $\mathbf{x}_i$ is the descriptor vector of molecule i, including the intercept term. The warning leverage was defined as:

$$\text{h}^* = \frac{3(p+1)}{n} \tag{S18}$$

where n is the number of molecules and p is the number of descriptors excluding the intercept. Williams plots of internally studentized residuals against leverage values were used to assess the applicability domain of each model.

S1.9 Bond dissociation enthalpy calculations

The parent molecules and the corresponding radical fragments were treated using the same geometry-optimization, harmonic-frequency, and single-point-energy levels described in Section S1.3. Open-shell radical fragments were calculated using unrestricted density functional theory. Enthalpies at 298.15 K were obtained by combining the refined single-point electronic energies with the thermal enthalpy corrections derived from the frequency calculations.

For each molecule included in the BDE analysis, all distinct C–C and C–H bonds were considered. For C–C bond homolysis, the bond dissociation enthalpy was calculated as:

$$\text{BDE}_{\text{C-C}} = \text{H(R}\cdot\text{)+H(R}'\cdot\text{)-H(R-R}'\text{)} \tag{S19}$$

where H(R-R') is the enthalpy of the molecule, and H(R·), H(R'·) are the enthalpies of radical fragments.

For C–H bond homolysis, the bond dissociation enthalpy was calculated as:

$$\text{BDE}_{\text{C-H}} = \text{H(R}\cdot\text{)+H(H}\cdot\text{)-H(R-H)} \tag{S20}$$

where H(R−H) is the enthalpy of the parent molecule, H(R·) is the enthalpy of the H-abstraction radical, and H(H·) is the enthalpy of the hydrogen atom.

The minimum C–C and C–H BDEs were used as comparative thermodynamic descriptors of susceptibility to initial C–C bond homolysis and H abstraction, respectively. These descriptors do not account for kinetic barriers,

subsequent radical reactions, oxygen transport, or condensed-phase operating conditions and were therefore not interpreted as complete measures of fuel stability.

S1.10. Validation of the computational workflow

The computational workflow was quantitatively validated against available experimental data for representative cyclic hydrocarbons. The validation included liquid density, gravimetric net heat of combustion (NHOC), viscosity, and flash point, which represent the major calculated properties used in the structure–property analysis and multi-objective screening. Benchmark compounds were selected based on the availability of experimental data with comparable property definitions, physical states, temperatures, and measurement conditions.

For density and viscosity, each benchmark compound was calculated at the temperature corresponding to its experimental measurement. The agreement between calculated and experimental values was evaluated using the number of validation points (n), mean absolute error (MAE), mean absolute percentage error (MAPE), root-mean-square error (RMSE), coefficient of determination (R^2), and mean signed error (bias). Volumetric NHOC was not independently validated because it was derived from the calculated density and gravimetric NHOC values. Specific impulse was not included in the statistical validation because it is not an intrinsic molecular property and depends strongly on propulsion-system parameters; however, identical RPA conditions were applied to all candidate fuels to ensure internally consistent comparison.

The obtained validation errors were considered when interpreting candidates located close to the viscosity and flash-point screening boundaries. Therefore, the calculated properties were treated as predictive estimates for comparative screening rather than direct substitutes for experimental measurements.

S1.10.1 Validation of liquid density

The computational protocols for liquid density, gravimetric net heat of combustion (NHOC), dynamic viscosity, and flash point were evaluated against experimental data for representative hydrocarbons. Before statistical analysis, the benchmark data were examined for consistency in property definition, physical state, units, temperature, and relevant measurement conditions. Density values were compared at 298.15 K. Gravimetric NHOC values were expressed on the same liquid-fuel net-heat basis for complete combustion to CO_2(g) and H_2O(g). Only dynamic viscosity was included, and each calculated viscosity was compared with the corresponding experimental value at the same temperature.

For experimental values reported as a range or with an uncertainty, the central value was used in the statistical analysis. The signed prediction error was defined as:

$$e_i = y_{\mathrm{calc},i} - y_{\mathrm{exp},i} \quad \text{(S21)}$$

and the predictive errors were evaluated using the mean absolute error (MAE), mean absolute percentage error (MAPE), root-mean-square error (RMSE), mean signed error (bias), and maximum absolute error (MaxAE):

$$\mathrm{MAE}=\frac{1}{n}\sum_{i=1}^{n}|e_i| \tag{S22}$$

$$\mathrm{MAPE}=\frac{100}{n}\sum_{i=1}^{n}\left|\frac{e_i}{y_{\exp,i}}\right| \tag{S23}$$

$$\mathrm{RMSE}=\sqrt{\frac{1}{n}\sum_{i=1}^{n}e_i^2} \tag{S24}$$

$$\mathrm{Bias}=\frac{1}{n}\sum_{i=1}^{n}e_i \tag{S25}$$

$$\mathrm{MaxAE}=\max_{1\le i\le n}|e_i| \tag{S26}$$

Experimental–calculated trend agreement was additionally examined using ordinary least-squares regression,

$$y_{\mathrm{calc}}=a+b\,y_{\exp} \tag{S27}$$

where a, b, and R^2 are the intercept, slope, and coefficient of determination, respectively. The R^2 describes the linear agreement between calculated and experimental values, whereas MAE, RMSE, bias, and MaxAE were used to assess the magnitude and direction of the prediction errors.

S1.10.1 Validation of liquid density

Liquid density was evaluated using ten hydrocarbons for which experimental data at 298.15 K were available. The MD calculations were performed at the same temperature as the experimental measurements. The benchmark data are summarized in Table S1.3.

Table S1.3 Comparison of the calculated and experimental liquid densities.

Compound	Experimental density/g·cm^{-3}	Experimental temperature / K	Calculated density/g·cm^{-3}	Calculation temperature / K	Reference
trans-Decalin	0.866	298.15	0.864	298.15	[1]
cis-Decalin	0.893	298.15	0.892	298.15	[1]
JP-10	0.932	298.15	0.937	298.15	[2]
Cyclohexane	0.774	298.15	0.770	298.15	[3]
Methylcyclohexane	0.765	298.15	0.762	298.15	[4]
Ethylcyclohexane	0.784	298.15	0.782	298.15	[5]
n-Butylcyclohexane	0.796	298.15	0.795	298.15	[6, 7]
Quadricyclane	0.974	298.15	0.972	298.15	[8]

Dicyclohexyl	0.883	298.15	0.878	298.15	[3]
1,3-Dimethyladamantane	0.898	298.15	0.896	298.15	[9]

Table S1.4 Statistical performance of the liquid-density calculations.

Number of validation points, n	10
MAE / g·cm^{-3}	0.003
MAPE / %	0.316
RMSE / g·cm^{-3}	0.003
Bias / g·cm^{-3}	−0.002
MaxAE / g·cm^{-3}	0.005
R^2	0.999
Slope	1.014
Intercept / g·cm^{-3}	−0.014

The liquid-density calculations showed small absolute and relative deviations from the experimental values, with an MAE of 0.003 g·cm^{-3}, an RMSE of 0.003 g·cm^{-3}, and a MAPE of 0.316%. The maximum absolute deviation was 0.0050 g·cm^{-3}. The small negative bias (−0.002 g·cm^{-3}) indicates only a minor overall tendency toward underprediction. The parity regression gave R^2=0.999 with a slope of 1.0144, indicating that the calculated values reproduce the experimental variation across the benchmark set.

S1.10.2 Validation of gravimetric net heat of combustion

Gravimetric NHOC was evaluated using ten liquid hydrocarbons. Experimental and calculated values were placed on the same net-heat-of-combustion basis, with complete combustion to CO_2(g) and H_2O(g). All NHOC values are reported as positive magnitudes of the heat released during combustion. The benchmark data are listed in Table S1.5.

Table S1.5 Comparison of experimental and calculated gravimetric NHOC.

Compound	Experimental NHOC / MJ·kg^{-1}	Calculated NHOC / MJ·kg^{-1}	Reference
trans-Decalin	42.54	41.88	[10]
cis-Decalin	42.62	41.94	[10]
JP-10	42.18	42.04	[11]
Cyclohexane	43.42	42.97	[12]

Methylcyclohexane	43.36	42.85	[12]
Ethylcyclobutane	44.59	43.68	[13]
n-Butylcyclohexane	43.42	42.94	[12]
Quadricyclane	44.35	43.97	[14]
Dicyclohexyl	42.73	42.19	[15]
1,3-Dimethyladamantane	41.72	41.47	[16]

Table S1.6 Statistical performance of the gravimetric NHOC calculations.

Number of validation points, n	10
MAE / MJ·kg^{-1}	0.500
MAPE / %	1.156
RMSE / MJ·kg^{-1}	0.542
Bias / MJ·kg^{-1}	−0.500
MaxAE / MJ·kg^{-1}	0.910
R^2	0.947
Slope	0.876
Intercept / MJ·kg^{-1}	4.833

The gravimetric NHOC calculations gave an MAE of 0.500 MJ·kg^{-1}, an RMSE of 0.542 MJ·kg^{-1}, and a MAPE of 1.156%, with a maximum absolute deviation of 0.910 MJ·kg^{-1}. The negative bias of −0.500 MJ·kg^{-1} indicates an overall tendency toward underprediction. The parity regression gave R^2=0.947, showing that the overall experimental variation is retained. Because the benchmark compounds span a relatively narrow NHOC range, the absolute error metrics provide a more direct measure of predictive accuracy than R^2 alone.

S1.10.3 Validation of viscosity

Dynamic viscosity was evaluated using 13 condition-matched experimental–calculated pairs. Three data points were evaluated at 298.15 K and ten at 233.15 K. Each calculated value was compared with the experimental viscosity at the same temperature. For experimental values reported as a range or with an uncertainty, the central value was used for calculation of the statistical metrics. The complete dataset is listed in Table S1.7.

Table S1.7 Comparison of experimental and calculated viscosity.

Compound	Experimental viscosity / mPa·s	Experimental temperature / K	Calculated viscosity / mPa·s	Calculation temperature / K	Reference
trans-Decalin	1.956	298.15	1.136	298.15	[1]
cis-Decalin	2.901	298.15	1.186	298.15	[1]
JP-10	2.767–2.782	298.15	1.120	298.15	[17]
JP-10	17.70	233.15	16.314	233.15	[17]
Cyclopentane	0.988	233.15	0.853	233.15	[18]
Ethylcyclohexane	5.470	233.15	4.583	233.15	[19]
6-Methyltricyclo[3.2.1.0^{2,4}]octane	10.5±0.5	233.15	8.973	233.15	[20]
Spiro[cyclopropane-1,6′-tricyclo[3.2.1.0^{2,4}]octane]	12.9±0.6	233.15	10.759	233.15	[20]
spiro[cyclopropane-1,2′-norbornane]	6.0±0.3	233.15	4.953	233.15	[20]
endo-(1S,2R,4R)-2-Propylbicyclo[2.2.1]heptane	9.950	233.15	7.536	233.15	[21]
endo-(1S,2R,4R)-2-Butylbicyclo[2.2.1]heptane	18.463	233.15	15.737	233.15	[21]
endo-(1S,2R,4R)-2-Pentylbicyclo[2.2.1]heptane	31.058	233.15	27.682	233.15	[21]
endo-(1S,2R,4R)-2-Hexylbicyclo[2.2.1]heptane	48.636	233.15	44.638	233.15	[21]

Table S1.8 Statistical performance of the viscosity.

Metric	All condition-matched data	233.15 K subset
Number of validation points, n	13	10
MAE / mPa·s	1.833	1.964
MAPE / %	23.468	14.442
RMSE / mPa·s	2.107	2.267
Bias / mPa·s	−1.833	−1.964

MaxAE / mPa·s	3.998	3.998
R^2	0.998	0.998
Slope	0.931	0.925
Intercept / mPa·s	−0.938	−0.754

For the complete viscosity dataset, the MAE and RMSE were 1.833 and 2.107 mPa·s, respectively. Because the subsequent screening uses viscosity at 233.15 K, the ten-point subset at this temperature was also evaluated separately, giving an MAE of 1.964 mPa·s and an RMSE of 2.267 mPa·s. The parity regression for the 233.15 K subset gave R^2=0.998, indicating that the relative variation among the benchmark compounds is well reproduced. The negative bias of −1.964 mPa·s indicates an overall tendency toward underprediction at this temperature. The 233.15 K RMSE was subsequently used in the uncertainty analysis in Section S5.3.

S1.10.4 Validation of flash point

Flash point was evaluated using ten hydrocarbons and the corresponding references and test information are given in Table S1.9 where available.

Table S1.9 Comparison of experimental and calculated flash point.

Compound	Experimental flash point / K	Calculated flash point / K	Reference
trans-Decalin	326.85	326.48	[1]
cis-Decalin	332.65	331.38	[1]
JP-10	327.55	325.84	[22]
Cyclohexane	253.15	251.06	[23]
Methylcyclohexane	269.3	267.47	[23]
Ethylcyclohexane	295.15	293.06	[23]
n-Butylcyclohexane	324.70	322.74	[6, 7]
Quadricyclane	284.15	281.95	[8]
Dicyclohexyl	364.70	362.75	[8]
1,3-Dimethyladamantane	325.15	322.89	[23]

Table S1.10 Statistical performance of the flash point.

Number of validation points, n	10
MAE / K	1.773
MAPE / %	0.584

RMSE / K	1.853
Bias / K	−1.773
MaxAE / K	2.26
R^2	0.9997
Slope	1.005
Intercept / K	−3.349

The flash-point calculations gave an MAE of 1.773 K and an RMSE of 1.853 K, corresponding to a MAPE of 0.584% and a maximum absolute deviation of 2.260 K. The parity regression gave R^2=0.9997 with a slope of 1.005, indicating that the variation in the experimental flash points is closely reproduced across the benchmark set. The negative bias of −1.773 K indicates an overall tendency toward underprediction. The resulting RMSE was subsequently used in the uncertainty analysis in Section S5.3.

As shown in Figure S1.1, the validation results quantify the prediction errors associated with the four property-calculation protocols. Density and flash point show relatively small deviations from experiment, while the NHOC and viscosity calculations exhibit systematic underprediction to different extents. These validation results provide the error scales used in the subsequent interpretation of the calculated properties; the screening implications of the viscosity and flash-point uncertainties are examined separately in Section S5.3.

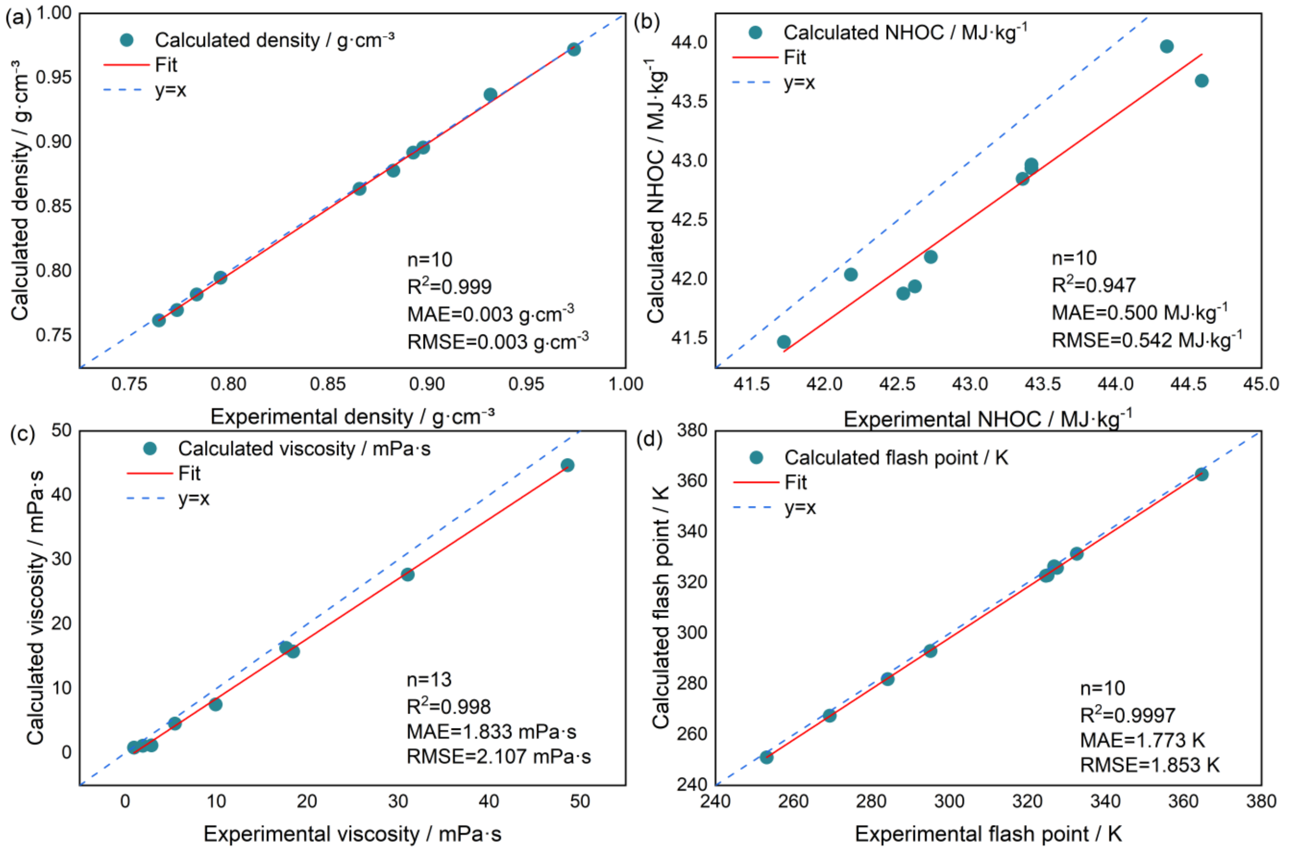

Figure S1.1 Experimental–calculated parity plots for (a) liquid density, (b) gravimetric NHOC, (c) dynamic viscosity, and (d) flash point. The dashed diagonal represents $y_{calc}=y_{exp}$, and the solid line represents the OLS

regression.

S2 Density and packing efficiency

S2.1 Descriptor selection and definitions for the V_m/M model

To express density in a mass-normalized volume form, V_m/M, which is equal to $1/\rho$, was used as the response variable. A lower V_m/M therefore corresponds to a higher density. Structural descriptors, including alkyl chain length (N_C), substitution position (I_α), branching (I_{iso}), cycloalkyl ring type (I_{Cycle3}, I_{Cycle4}, I_{Cycle5}, I_{Cycle6}, I_{Cnor}), and connection topology (I_{Fused}, I_{Spiro}), were introduced to describe the remaining structural variations. The common reference structure was β-n-C1, for which all structural descriptors were set to 0. N_C was used only for alkyl-substituted derivatives and was defined as the carbon number increment relative to C1. For cycloalkyl-substituted derivatives, $N_C = 0$ and $I_{iso} = 0$. Linked topology was used as the reference level, therefore, $I_{Fused} = 0$ and $I_{Spiro} = 0$ correspond to linked topology within the cycloalkyl subset. The definitions and coding schemes of all descriptors are summarized in Table S2.1.

Table S2.1 Definition of descriptors used in the V_m/M model.

Descriptor	Symbol	Description
Alkyl chain length	N_C	Carbon-number increment of alkyl substituent relative to C1; C1 = 0, C2 = 1, ..., C6 = 5; set to 0 for cycloalkyl-substituted derivatives
Substitution position	I_α	α = 1, β = 0
Branching type	I_{iso}	iso-alkyl = 1, n-alkyl = 0; set to 0 for cycloalkyl-substituted derivatives
Three-membered ring	I_{Cycle3}	Cycle3 = 1, otherwise 0
Four-membered ring	I_{Cycle4}	Cycle4 = 1, otherwise 0
Five-membered ring	I_{Cycle5}	Cycle5 = 1, otherwise 0
Six-membered ring	I_{Cycle6}	Cycle6 = 1, otherwise 0
Norbornane substitution	I_{Cnor}	Cnor = 1, otherwise 0
Fused topology	I_{Fused}	Fused topology = 1, otherwise 0
Spiro topology	I_{Spiro}	Spiro topology = 1, otherwise 0

S2.2 Statistical analysis of the V_m/M regression model

An MLR model was constructed to correlate V_m/M with structural descriptors using ordinary least squares. The model was developed based on a dataset of 50 molecules. The corresponding regression equation is:

$$V_m/M = 1.155-0.001N_C-0.007I_\alpha-0.026I_{iso}-0.051I_{Cycle3}-0.073I_{Cycle4}-0.068I_{Cycle5}$$

$-0.074I_{Cycle6}-0.123I_{Cnor}-0.006I_{Fused}-0.011I_{Spiro}$

The regression coefficients, multicollinearity diagnostics, model-performance metrics, residual analysis, outlier identification, and leverage-based applicability-domain assessment are summarized in Tables S2.2–S2.3 and Figure S2.1.

Table S2.2 Regression coefficients and multicollinearity diagnostics of the V_m/M model.

Descriptor	β	Standardized β	Std. Error	t-value	p-value	VIF
Intercept	1.155	—	0.003	363.054	<0.001	—
N_C	-0.001	-0.041	0.001	-1.001	0.323	3.349
I_α	-0.007	-0.083	0.002	-3.706	<0.001	1.000
I_{iso}	-0.026	-0.235	0.003	-8.384	<0.001	1.549
I_{Cycle3}	-0.051	-0.408	0.004	-11.678	<0.001	1.510
I_{Cycle4}	-0.073	-0.585	0.004	-16.773	<0.001	1.510
I_{Cycle5}	-0.068	-0.546	0.004	-15.639	<0.001	1.510
I_{Cycle6}	-0.074	-0.595	0.004	-17.056	<0.001	1.510
I_{Cnor}	-0.123	-0.980	0.004	-28.091	<0.001	1.510
I_{Fused}	-0.006	-0.055	0.003	-1.944	0.059	1.600
I_{Spiro}	-0.011	-0.108	0.003	-3.784	<0.001	1.600

Table S2.3 Statistical performance of the V_m/M model.

Metric	Value
R^2	0.980
Adjusted R^2	0.975
RMSE	0.006
MAE	0.004
Q^2	0.967
F-statistic	193.6
p-value	< 0.001

The V_m/M regression model shows good statistical performance, with adjusted $R^2 = 0.975$, cross-validated $Q^2 = 0.967$, and RMSE = 0.006 $cm^3 \cdot g^{-1}$. Most structural descriptors are statistically significant, except for N_C and I_{Fused}. The non-significant coefficient of N_C indicates that normal alkyl chain extension has only a weak effect on mass-normalized volume, whereas the slightly non-significant I_{Fused} coefficient suggests that the fused-topology effect relative to linked topology is weaker than the spiro-topology effect. All VIF values are below 5, indicating acceptable multicollinearity among the selected descriptors.

Residual analysis, outlier identification, and leverage-based applicability-domain assessment were further performed to evaluate whether the model predictions involved influential response outliers or extrapolation beyond the investigated structural space. For the V_m/M model, p = 10 descriptors and n = 50 molecules were used; therefore, the warning leverage threshold was:

$$h^* = \frac{3(p+1)}{n} = \frac{3(10+1)}{50} = 0.660 \quad \text{(S28)}$$

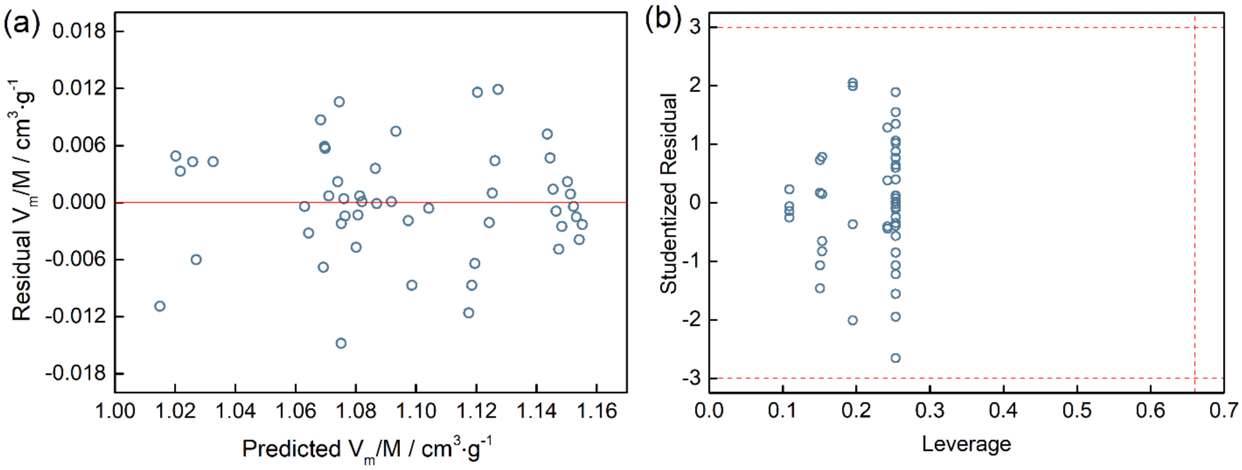

Figure S2.1. Residual and applicability-domain analysis of the V_m/M regression model.

(a) Residuals versus predicted V_m/M values. (b) Williams plot based on studentized residuals and leverage values. The vertical dashed line indicates the warning leverage threshold h^* = 0.660, and the horizontal dashed lines indicate studentized residuals of ±3.

As shown in Figure S2.1, the residuals are generally distributed around zero without an obvious systematic trend. Molecules with $|r_i|>3$ were treated as potential response outliers, whereas molecules with $h_i>h^*$ were considered potentially outside the applicability domain. No molecule showed $|r_i|>3$, and all leverage values were below h^* = 0.660. The largest studentized residual was observed for β-F-Cycle6 (r_i = -2.653, h_i = 0.253), which remained within the accepted residual and leverage ranges. Therefore, no obvious response outlier or applicability-domain outlier was identified for the V_m/M model. Overall, the model was interpreted within the structural space covered by the present decalin derivative library.

S2.3 Definition and calculation of microstructural descriptors

For each molecule, the molar volume was obtained from the calculated density according to:

$$V_m = \frac{M}{\rho} \quad \text{(S29)}$$

where M is the molecular mass and ρ is the calculated density. The V_m/M used in the MLR model is $1/\rho$. The intrinsic molecular volume was approximated by the 0.001 a.u. electron-density isosurface volume, denoted as V_{int}. When the volume was first obtained in molecular units ($Å^3$), it was converted into molar units ($cm^3 \cdot mol^{-1}$) according to:

$$V_{int}(cm^3 \cdot mol^{-1}) = V_{int}(Å^3) \times N_A \times 10^{-24} \tag{S30}$$

where N_A is Avogadro's constant. The intermolecular free volume was then defined as:

$$V_{free} = V_m - V_{int} \tag{S31}$$

To express the intrinsic and free-volume contributions on a mass-normalized basis, V_{int} and V_{free} were analyzed as V_{int} /M and V_{free} /M, respectively. In the present work, the selected microstructural descriptors were used as structural proxies for the two volume contributions. Specifically, R_g was used to characterize intrinsic compactness and was therefore mainly associated with V_{int}, whereas χ_{asph}, $\ln(N_{conf}+1)$ and $\sigma(V)$ were used to describe packing compatibility in terms of shape compatibility, conformational adjustability, and conformational dispersion, respectively, and were therefore mainly associated with $V_{free.}$

S2.3.1 Radius of gyration (R_g)

The R_g describes the mass-weighted spatial distribution of atoms within a molecule and was used here as a geometric descriptor of molecular spatial span. Smaller R_g values correspond to a more compact molecular arrangement and therefore a smaller intrinsic occupied volume.

$$R_g = \sqrt{\frac{\sum_i m_i \, |\mathbf{r}_i - \mathbf{r}_{cm}|^2}{\sum_i m_i}} \tag{S32}$$

where m_i and r_i are the atomic mass and position vector of atom i, respectively, and r_{cm} is the center-of-mass position. R_g was calculated using Multiwfn based on DFT-optimized geometries. For flexible molecules, R_g values were averaged over the conformational ensemble obtained from CREST sampling using Boltzmann weighting.

S2.3.2 Molecular asphericity (χ_{asph})

In the present work, χ_{asph} was used to characterize shape compatibility for intermolecular packing.

$$\chi_{asph} = \frac{S^3}{36\pi V^2} - 1 \tag{S33}$$

where S and V are the molecular surface area and molecular volume, respectively. For a perfect sphere, $\chi_{asph} = 0$; larger χ_{asph} values indicate a greater deviation from spherical symmetry and thus a more anisotropic molecular shape. Such shapes are generally less favorable for efficient intermolecular fitting and tend to be associated with a larger intermolecular free-volume contribution. The molecular surface area and volume were calculated using Multiwfn based on the DFT-optimized geometries. For flexible molecules, χ_{asph} values were averaged over the conformational ensemble obtained from CREST sampling using Boltzmann weighting.

S2.3.3 Number of low-energy accessible conformers (N_{conf})

To characterize conformational adjustability, the number of low-energy accessible conformers, N_{conf} was introduced. N_{conf} was defined as the number of non-duplicated conformers retained within the selected energy window from CREST conformational sampling. A larger N_{conf} indicates that the molecule can access a larger number of low-energy spatial arrangements and therefore has greater conformational adjustability. Because the raw conformer counts were highly skewed, the quantity was transformed as:

$$\ln(N_{conf}+1) \tag{S34}$$

Because the raw conformer counts were highly skewed, $\ln(N_{conf}+1)$ was used to reduce skewness and limit the influence of molecules with unusually large conformer counts. Larger values indicate greater conformational accessibility. Larger $\ln(N_{conf}+1)$ values correspond to greater conformational adjustability. In the context of liquid packing, this descriptor was used together with σ(V) to characterize the conformational contribution to packing compatibility.

S2.3.4 Conformational volume fluctuation (σ(V))

σ(V) is defined as the Boltzmann-weighted standard deviation of molecular volumes across the conformational ensemble and serves as a quantitative descriptor of conformational variability. Larger σ(V) values indicate that the accessible conformers differ more strongly in their spatial organization, which is generally less favorable for uniform and efficient packing.

$$\sigma(V) = \sqrt{\frac{1}{N}\sum_{i=1}^{N}(V_i-\bar{V})^2} \tag{S35}$$

where V_i is the 0.001 a.u. electron-density isosurface volume of conformer i, and $\bar{V}$ is the average volume of all conformers. Conformational sampling was performed using CREST at the GFN2-xTB level within an energy window of 3.0 kcal·mol^{-1}. For each conformer, the molecular volume was calculated using Multiwfn. The ensemble-averaged standard deviation was then computed. For molecules represented by a single conformer, σ(V) = 0.

S2.3.5 Correlation of microstructural descriptors with V_{int} and V_{free}

To examine the physical interpretation of the selected descriptors, their relationships with V_{int} and V_{free} were further analyzed. As shown in Figure S2.2(a), V_{int} generally increases with increasing R_g, indicating that a larger molecular spatial span enlarges the intrinsic occupied volume. This trend supports the use of R_g as a geometric descriptor of molecular extension. Figure S2.2(b) shows that V_{free} generally increases with increasing χ_{asph}, indicating that more anisotropic molecular shapes tend to reduce packing efficiency and increase the free volume

contribution. In addition, the two-dimensional conformational map defined by $\ln(N_{conf}+1)$ and $\sigma(V)$ shows that larger V_{free} values are preferentially distributed in regions of greater conformational adjustability and/or stronger conformational dispersion. These observations support the use of χ_{asph}, $\ln(N_{conf}+1)$ and $\sigma(V)$ as descriptors of packing compatibility.

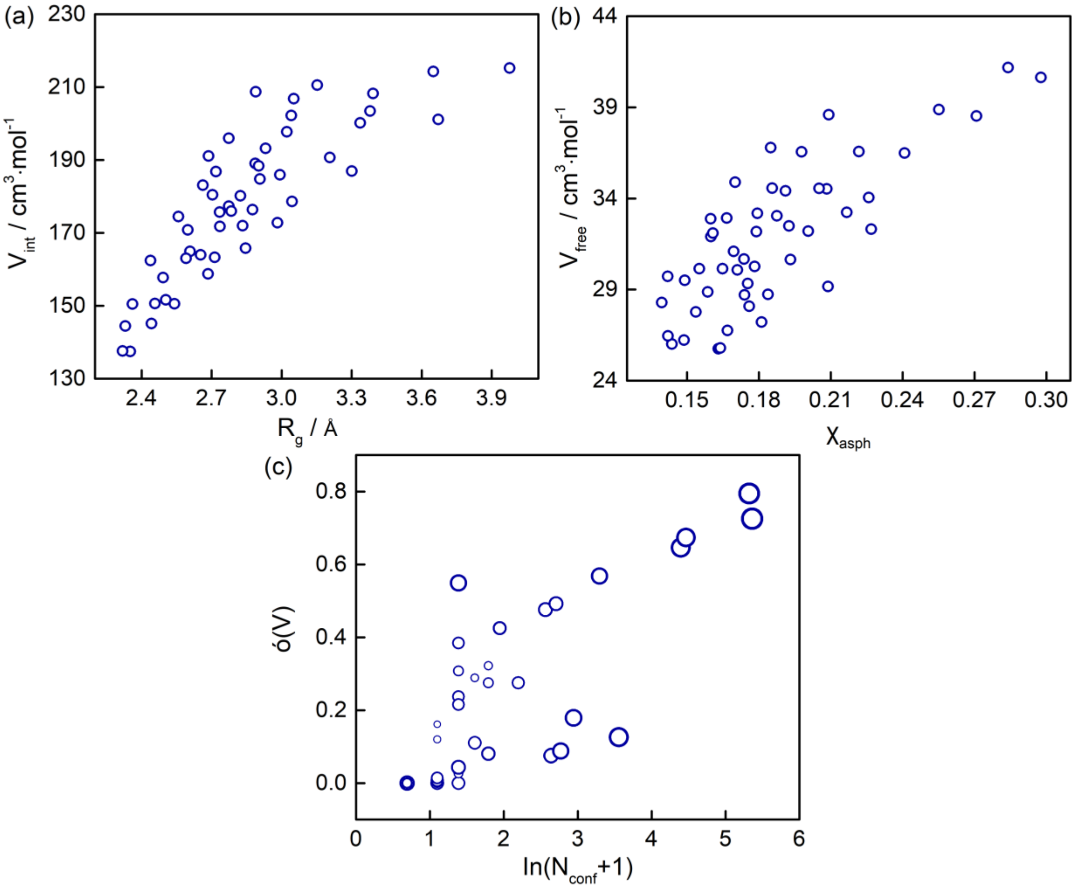

Figure S2.2 Correlation of the selected microstructural descriptors and volume components.

(a)Correlation between V_{int} and R_g for the substituted trans-decalin derivatives.

(b) Relationship between V_{free} and χ_{asph} for the substituted trans-decalin derivatives.

(c) Two-dimensional conformational map defined by $\ln(N_{conf}+1)$ and $\sigma(V)$, in which V_{free} is encoded by bubble size.

S2.3.6 Mass-corrected residual analysis of microstructural descriptors

For comparisons involving cycloalkyl topologies and ring types, some geometry- and conformation-related descriptors may vary with molecular mass. Therefore, mass-corrected residuals were calculated to distinguish structural effects from the general mass dependence. Each descriptor D was fitted against molar mass according to:

$$D_{fit} = a + bM \quad \text{(S36)}$$

The mass-corrected residual was then calculated as:

$$D_{res} = \text{D-(a+bM)} \tag{S37}$$

Positive residuals indicate descriptor values larger than expected at a given molar mass, whereas negative residuals indicate smaller values than expected. This analysis was applied to R_g, χ_{asph}, ln(N_{conf}+1), and σ(V).

Table S2.4 Linear equations used to calculate mass-corrected descriptor residuals.

Descriptor D	Fitting equation
R_g	R_g = 0.8132+0.01017M
χ_{asph}	X_{asph} = 0.040763+0.000707M
ln(N_{conf}+1)	ln(N_{conf}+1) = −0.7395+0.009525M
σ(V)	σ(V) = −0.2314+0.001653M

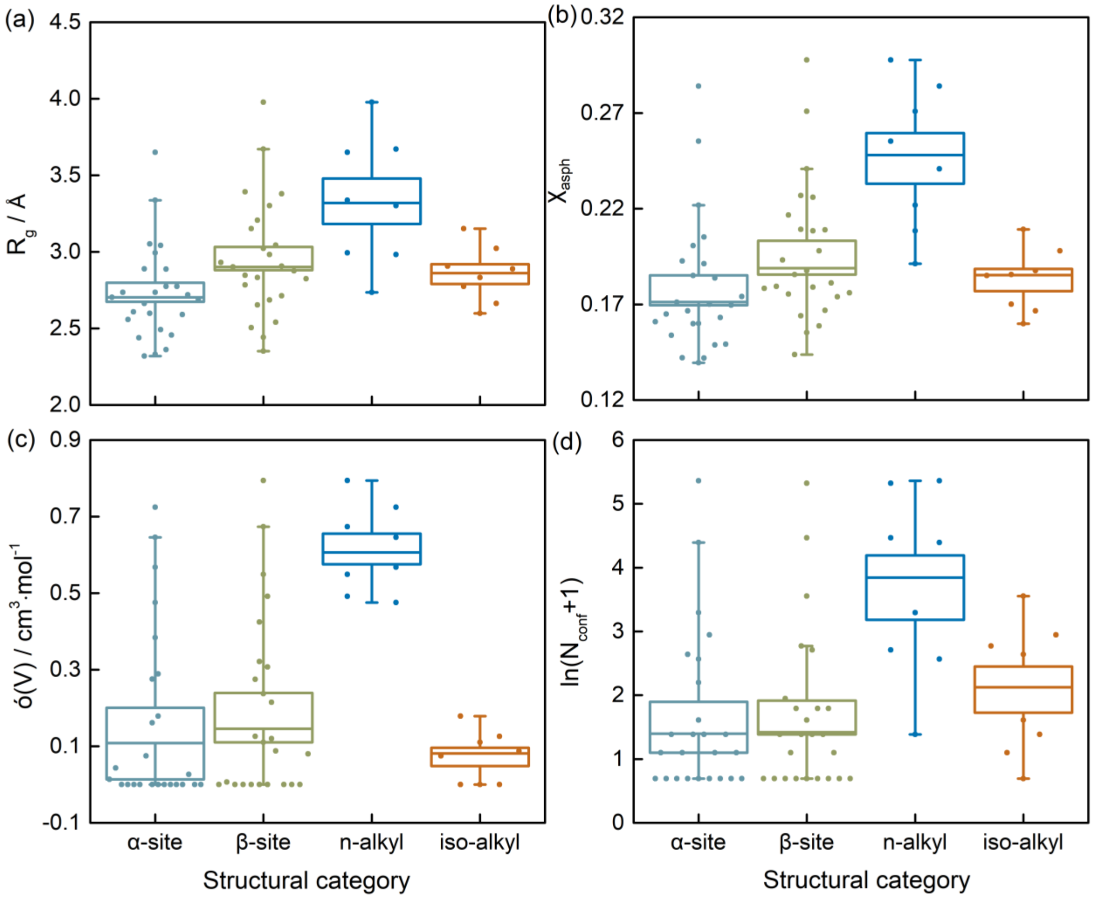

Figure S2.3 Mass-corrected residual distributions of microstructural descriptors.

(a-d) Distributions of R_g, χ_{asph}, ln(N_{conf}+1), and σ(V) grouped by alkyl-substituent class.

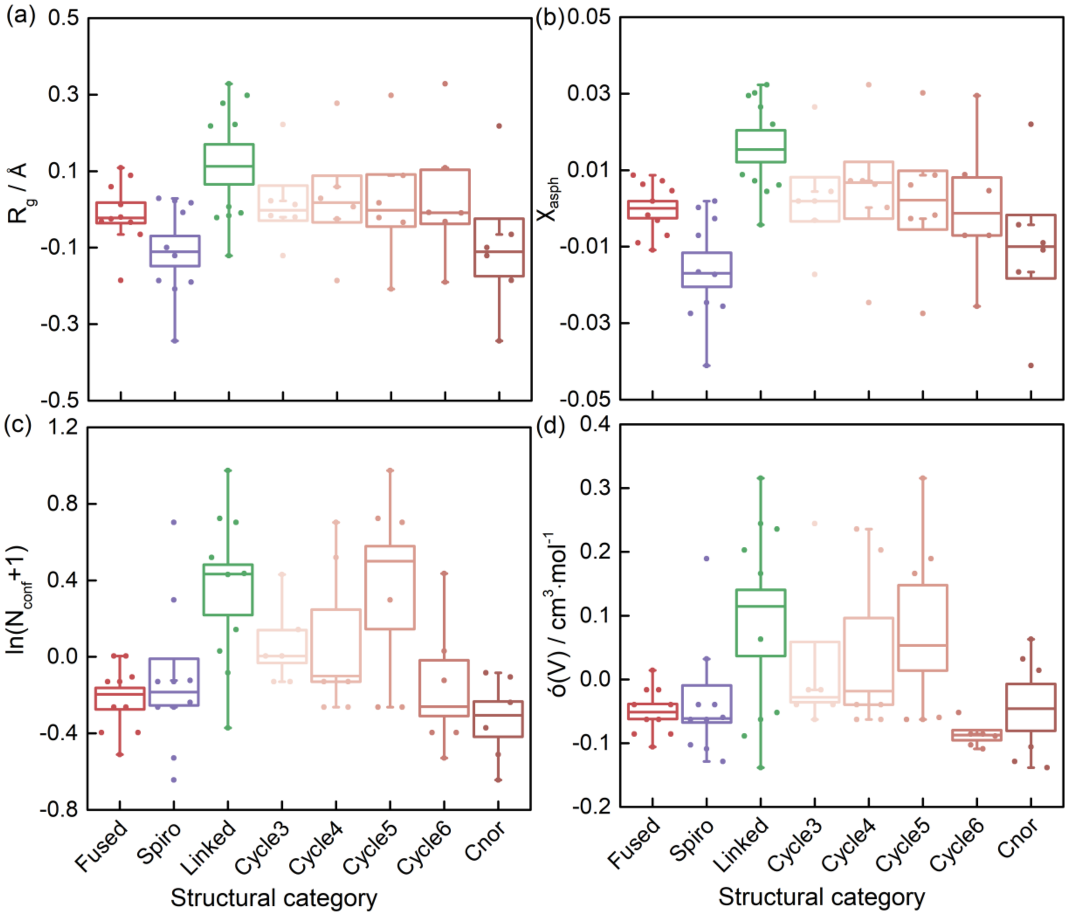

Figure S2.4 Mass-corrected residual distributions of microstructural descriptors.

(a-d) Residual distributions of R_g, χ_{asph}, ln(N_{conf}+1), and σ(V) grouped by cycloalkyl-substituent class.

S3 Energy and propulsion performance

S3.1 Descriptor selection and definitions for gravimetric NHOC and I_{sp} model

For the MLR analyses of gravimetric NHOC and I_{sp}, the descriptor set comprised H/C, E_{strain}, I_{α}, I_{iso}, I_{Fused}, I_{Spiro}, and I_{Linked}. H/C was used as the compositional descriptor, while ring type was represented through E_{strain} rather than separate categorical indicators. Substitution position, branching pattern, and connection topology were encoded by binary indicator variables. To account for the non-additive coupling between the high ring strain of cyclopropane and the local geometric constraint imposed by fused topology, a fused-cyclopropane interaction descriptor, $E_{strain}\times I_{Fused}\times I_{Cycle3}$, was further introduced. For alkyl-substituted derivatives, E_{strain}, I_{Fused}, I_{Spiro}, I_{Linked} and $E_{strain}\times I_{Fused}\times I_{Cycle3}$ were all coded as 0. For cycloalkyl-substituted derivatives, I_{iso} was coded as 0, E_{strain} was assigned according to ring type, and the corresponding topology indicator was set to 1 according to the connection mode.

E_{strain}×I_{Fused}×I_{Cycle3} is nonzero only for fused Cycle3 derivatives. The definitions and coding schemes of all descriptors are summarized in Table S3.1.

Table S3.1 Definition of descriptors used in the gravimetric NHOC and I_{sp} models.

Descriptor	Symbol	Description
Hydrogen-to-carbon ratio	H/C	Atomic H/C ratio, reflecting molecular hydrogen richness
Ring strain energy	E_{strain}	Ring-type strain energy (kcal mol^{-1}), the assigned values were: three-membered = 27.6, four-membered = 26.4, five-membered = 6.5, six-membered = 0.0, norbornyl-type = 17.2.
Fused-cyclopropane interaction	E_{strain}×I_{Fused}×I_{Cycle3}	Interaction descriptor describing the coupled effect of ring strain and fused topology in fused Cycle3 derivatives; nonzero only when I_{Fused} = 1 and I_{Cycle3} = 1.
Substitution position	I_{α}	α = 1, β = 0
Branching type	I_{iso}	iso-alkyl = 1, n-alkyl = 0; set to 0 for cycloalkyl-substituted derivatives
Fused topology	I_{Fused}	Fused topology = 1, otherwise 0
Spiro topology	I_{Spiro}	Spiro topology = 1, otherwise 0
Linked topology	I_{Linked}	Linked topology = 1, otherwise 0

S3.2 Statistical analysis of the gravimetric NHOC regression model

An MLR model was constructed to quantify the dependence of gravimetric NHOC on molecular composition, substitution position, branching, connection topology, ring strain, and the fused-cyclopropane strain–topology interaction. The model was fitted by ordinary least squares using the dataset of 50 molecules. The final regression equation is:

$$\text{Gravimetric NHOC} = 33.625+4.992(H/C)+0.054I_{\alpha}+0.027I_{iso}+0.511I_{Fused}+0.426I_{Spiro}+0.493I_{Linked}+0.023E_{strain} +0.030E_{strain}\times I_{Fused}\times I_{Cycle3}$$

The detailed regression results, model-performance metrics, residual analysis, outlier identification, and leverage-based applicability-domain assessment for the gravimetric NHOC model are summarized in Tables S3.2–S3.4 and Figure S3.1.

Table S3.2 Regression coefficients and multicollinearity diagnostics of the gravimetric NHOC model.

Descriptor	β	Standardized β	Std. Error	t-value	p-value	VIF
Intercept	33.625	—	0.551	61.016	<0.001	—
H/C	4.992	1.760	0.298	16.761	<0.001	12.371

I_{α}	0.054	0.106	0.015	3.553	0.001	1.000
I_{iso}	0.027	0.039	0.025	1.093	0.281	1.429
I_{Fused}	0.511	0.803	0.053	9.569	<0.001	7.900
I_{Spiro}	0.426	0.669	0.047	9.001	<0.001	6.205
I_{Linked}	0.493	0.775	0.042	11.644	<0.001	4.970
E_{strain}	0.023	1.014	0.001	19.772	<0.001	2.949
$E_{strain}\times I_{Fused}\times I_{Cycle3}$	0.030	0.631	0.002	18.288	<0.001	1.338

Table S3.3 Statistical performance of the gravimetric NHOC model.

Metric	Value
R^2	0.963
Adjusted R^2	0.956
RMSE	0.049
MAE	0.038
Q^2	0.942
F-statistic	135.102
p-value	<0.001

The gravimetric NHOC model shows substantially improved statistical performance, with $R^2 = 0.963$, $Q^2 = 0.942$, and RMSE = 0.049 $MJ\cdot kg^{-1}$. Compared with the additive model, the inclusion of $E_{strain}\times I_{Fused}\times I_{Cycle3}$ markedly improves the description of fused Cycle3 derivatives, indicating that the ring-strain contribution of fused cyclopropane motifs cannot be fully represented by independent additive E_{strain} and topology terms. The interaction descriptor is highly significant ($p<0.001$) and has a low VIF value of 1.338, indicating that it does not introduce problematic additional multicollinearity. The relatively high VIF of H/C reflects the inherent correlation between molecular composition and structural class in the designed decalin derivative library; individual coefficient magnitudes were therefore interpreted with caution and considered together with cross-validation, residual analysis, and applicability-domain assessment.

For the gravimetric NHOC model, p = 8 descriptors and n = 50 molecules were used; therefore, the warning leverage threshold was:

$$h^* = \frac{3(p+1)}{n} = \frac{3(8+1)}{50} = 0.540 \quad \text{(S38)}$$

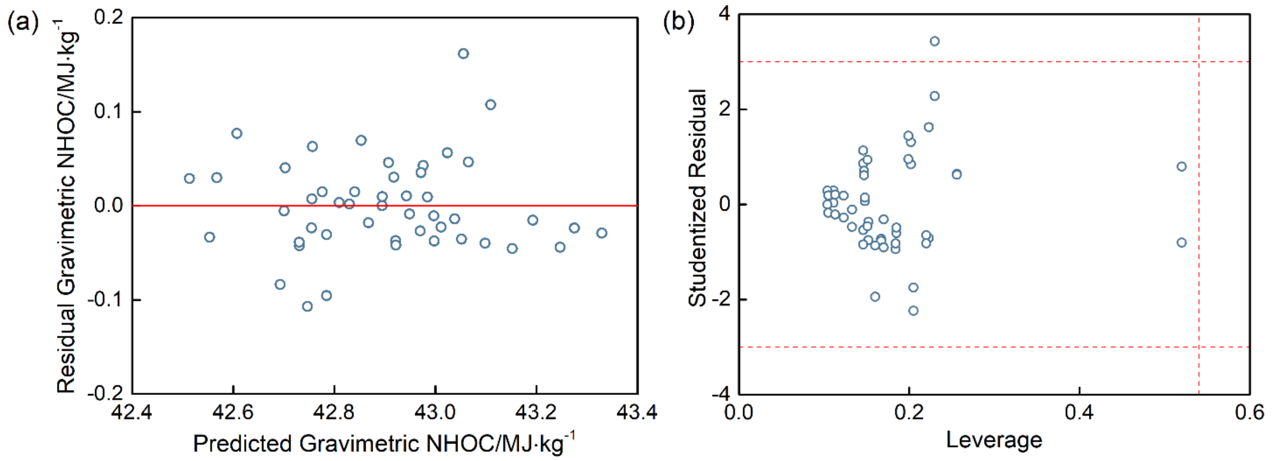

Figure S3.1. Residual and applicability-domain analysis of the gravimetric NHOC model.

(a) Residuals versus predicted gravimetric NHOC values. (b) Williams plot based on studentized residuals and leverage values. The vertical dashed line indicates the warning leverage threshold $h^* = 0.540$, and the horizontal dashed lines indicate studentized residuals of ±3.

As shown in Figure S3.1, the residuals are generally distributed around zero without an obvious systematic trend. Molecules with $|r_i|>3$ were treated as potential response outliers, whereas molecules with $hi>h^*$ were considered potentially outside the applicability domain. The largest studentized residual was observed for β-F-Cycle4 ($r_i = 3.433$, $h_i = 0.230$), indicating a potential response outlier. However, its leverage value was much lower than $h^* = 0.540$, indicating that β-F-Cycle4 is not an applicability-domain outlier. All molecules showed leverage values below h^*. Therefore, no applicability-domain outlier was identified for the gravimetric NHOC model, and the model was interpreted within the decalin-based structural space covered by the present molecular library.

Table S3.4. Largest residuals and applicability-domain assessment for the gravimetric NHOC model.

Molecule	Calculated $MJ \cdot kg^{-1}$	Predicted $MJ \cdot kg^{-1}$	Residual $MJ \cdot kg^{-1}$	Studentized residual	Leverage
β-F-Cycle4	43.2171	43.0553	0.1618	3.433	0.230
α-F-Cycle4	43.2168	43.1093	0.1075	2.281	0.230

α-F-Cycle6	42.6397	42.7467	-0.1070	-2.234	0.205

S3.3 Statistical analysis of the I_{sp} regression model

As with gravimetric NHOC, an MLR model was built to quantify the dependence of I_{sp} on molecular composition, ring strain energy, and structural features. The model was fitted by ordinary least squares to the same dataset of 50 molecules. The corresponding regression equation is:

$$I_{sp} = 348.043+8.161(H/C)+0.229I_{\alpha}+0.119I_{iso}+2.140I_{Fused}+1.778I_{Spiro}+2.063I_{Linked}+0.126E_{strain}\times I_{Fused}\times I_{Cycle3}+0.097E_{strain}$$

The detailed regression results, model-performance metrics, residual analysis, outlier identification, and leverage-based applicability-domain assessment for the I_{sp} model are summarized in Tables S3.5–S3.7 and Figure S3.2.

Table S3.5 Regression coefficients and multicollinearity diagnostics of the I_{sp} model.

Descriptor	β	Standardized β	Std. Error	t-value	p-value	VIF
Intercept	348.043	—	2.358	147.607	<0.001	—
H/C	8.161	0.458	1.274	6.404	<0.001	12.371
I_{α}	0.229	0.072	0.065	3.527	0.001	1.000
I_{iso}	0.119	0.027	0.106	1.121	0.269	1.429
I_{Fused}	2.140	0.536	0.228	9.371	<0.001	7.900
I_{Spiro}	1.778	0.445	0.202	8.784	<0.001	6.205
I_{Linked}	2.063	0.517	0.181	11.387	<0.001	4.970
E_{strain}	0.097	0.687	0.005	19.666	<0.001	2.949
$E_{strain}\times I_{Fused}\times I_{Cycle3}$	0.126	0.428	0.007	18.170	<0.001	1.338

Table S3.6 Statistical performance of the I_{sp} model.

Metric	Value
R^2	0.983
Adjusted R^2	0.980
RMSE	0.208
MAE	0.161
Q^2	0.973
F-statistic	296.757
p-value	<0.001

The I_{sp} model also shows strong predictive performance, with $R^2 = 0.983$, $Q^2 = 0.973$, and RMSE = 0.208 s.

Both E_{strain} and E_{strain}×I_{Fused}×I_{Cycle3} are statistically significant, confirming that propulsion performance is strongly affected by ring strain and by the additional strain–topology coupling in fused cyclopropane motifs. Similar to the gravimetric NHOC model, the added interaction term has a low VIF value of 1.338, whereas the higher VIF of H/C mainly reflects the coupled variation of composition and structural class in the designed molecular library.

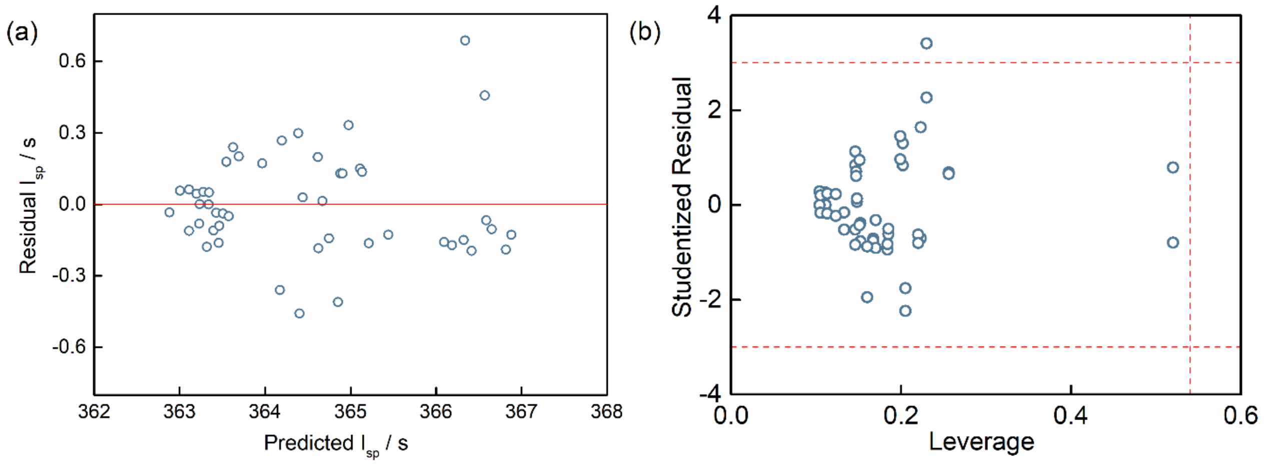

Figure S3.2. Residual and applicability-domain analysis of the I_{sp} model.

(a) Residuals versus predicted I_{sp} values. (b) Williams plot based on studentized residuals and leverage values. The vertical dashed line indicates the warning leverage threshold $h^* = 0.540$, and the horizontal dashed lines indicate studentized residuals of ±3.

For the I_{sp} model, the unified descriptor set was employed, resulting in $p = 8$, $n = 50$, and a warning leverage threshold $h^* = 0.540$. As shown in Figure S3.2, the residuals are generally centered around zero without an obvious systematic trend. The largest studentized residual was observed for β-F-Cycle4 ($r_i = 3.413$, $h_i = 0.230$), indicating a potential response outlier. However, its leverage value was much lower than $h^* = 0.540$, indicating that β-F-Cycle4 is not an applicability-domain outlier. All molecules showed leverage values below h^*. Therefore, no applicability-domain outlier was identified for the I_{sp} model, and the model was interpreted within the decalin-based structural space covered by the present molecular library.

Table S3.7. Largest residuals and applicability-domain assessment for the I_{sp} model.

Molecule	Calculated / s	Predicted / s	Residual / s	Studentized residual	Leverage

β-F-Cycle4	367.0281	366.3400	0.6881	3.413	0.230
α-F-Cycle4	367.0269	366.5693	0.4576	2.269	0.230
α-F-Cycle6	363.9452	364.4033	-0.4581	-2.236	0.205

S4 Viscosity and flash point

S4.1 Descriptor selection and definitions for η and T_f model

For the regression models of viscosity and flash point, M was retained as the mass-related descriptor. Structural effects were described using substitution position (I_α), branching type (I_{iso}), ring type indicators (I_{Cycle3}, I_{Cycle4}, I_{Cycle5}, I_{Cycle6}, I_{Cnor}), and topology indicators (I_{Fused}, I_{Spiro}). For cycloalkyl-substituted derivatives, linked topology was used as the reference level for topology, $I_{Fused} = 0$ and $I_{Spiro} = 0$ correspond to linked topology within the cycloalkyl subset. The definitions and coding schemes of all descriptors are summarized in Table S4.1.

Table S4.1 Definition of descriptors used in the ln(η) and T_f models.

Descriptor	Symbol	Description
Molar mass	M	Total molecular mass (g/mol)
Substitution position	I_α	α = 1, β = 0
Branching type	I_{iso}	iso-alkyl = 1, n-alkyl = 0; set to 0 for cycloalkyl-substituted derivatives
Three-membered ring	I_{Cycle3}	Cycle3 = 1, otherwise 0
Four-membered ring	I_{Cycle4}	Cycle4 = 1, otherwise 0
Five-membered ring	I_{Cycle5}	Cycle5 = 1, otherwise 0
Six-membered ring	I_{Cycle6}	Cycle6 = 1, otherwise 0
Norbornane substitution	I_{Cnor}	Cnor = 1, otherwise 0
Fused topology	I_{Fused}	Fused topology = 1, otherwise 0
Spiro topology	I_{Spiro}	Spiro topology = 1, otherwise 0

S4.2 Statistical analysis of the ln(η) regression model

To establish the structure-property relationship for viscosity, the natural logarithm of viscosity, ln(η), was chosen as the dependent variable to facilitate linearization of the data. The resulting regression equation is:

$$\ln(\eta) = -1.460+0.021M-0.075I_{\alpha}-0.025I_{iso}+0.577I_{Cycle3}+0.518I_{Cycle4}+0.607I_{Cycle5}$$
$$+0.785I_{Cycle6}+0.878I_{Cnor}-0.299I_{Fused}-0.154I_{Spiro}$$

The regression coefficients, multicollinearity diagnostics, model-performance metrics, residual analysis, outlier

identification, and leverage-based applicability-domain assessment are summarized in Tables S4.2–S4.3 and Figure S4.1.

Table S4.2 Regression coefficients and multicollinearity diagnostics of the ln(η) model.

Descriptor	β	Standardized β	Std. Error	t-value	p-value	VIF
Intercept	-1.460	—	0.254	-5.754	<0.001	—
M	0.021	0.736	0.001	15.926	<0.001	5.380
I_{α}	-0.075	-0.058	0.036	-2.101	0.042	1.990
I_{iso}	-0.025	-0.014	0.060	-0.409	0.685	1.755
I_{Cycle3}	0.577	0.291	0.072	8.038	<0.001	1.844
I_{Cycle4}	0.518	0.261	0.071	7.287	<0.001	1.916
I_{Cycle5}	0.607	0.306	0.075	8.071	<0.001	1.995
I_{Cycle6}	0.785	0.395	0.084	9.406	<0.001	2.081
I_{Cnor}	0.878	0.442	0.093	9.446	<0.001	2.159
I_{Fused}	-0.299	-0.186	0.068	-4.428	<0.001	2.023
I_{Spiro}	-0.154	-0.096	0.059	-2.599	0.013	2.006

Table S4.3 Statistical performance of the ln(η) model.

Metric	Value
R^2	0.970
Adjusted R^2	0.963
RMSE	0.111
MAE	0.074
Q^2	0.949
F-statistic	127.705
p-value	<0.001

The ln(η) model shows strong statistical performance, with $R^2 = 0.970$, adjusted $R^2 = 0.963$, $Q^2 = 0.949$, and RMSE = 0.111. The maximum VIF is 5.380 for M, slightly above the conventional threshold of 5 and indicating moderate rather than severe multicollinearity. This reflects the expected association between molecular mass and structural class in the designed library.

Residual analysis, outlier identification, and leverage-based applicability-domain assessment were further performed for the ln(η) model. For this model, $p = 10$ descriptors and $n = 50$ molecules were used; therefore, the warning leverage threshold was:

$$h^* = \frac{3(p+1)}{n} = \frac{3(10+1)}{50} = 0.660 \qquad (S39)$$

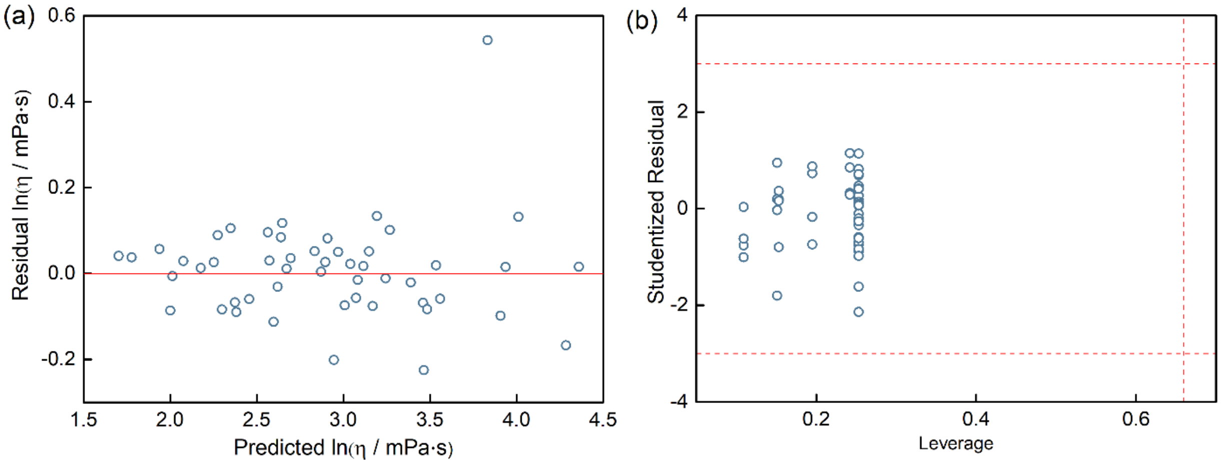

Figure S4.1. Residual and applicability-domain analysis of the ln(η) regression model.

(a) Residuals versus predicted ln(η) values. (b) Williams plot based on studentized residuals and leverage values. The vertical dashed line indicates the warning leverage threshold h^* = 0.660, and the horizontal dashed lines indicate studentized residuals of ±3.

As shown in Figure S4.1, most residuals are distributed around zero. Molecules with $|r_i|>3$ were treated as potential response outliers, whereas molecules with $h_i>h^*$ were considered potentially outside the applicability domain. The largest studentized residual was observed for α-S-Cnor (r_i = 4.921, h_i = 0.253), indicating that α-S-Cnor is a potential response outlier for the ln(η) model. However, its leverage value is much lower than h^* = 0.660, indicating that α-S-Cnor is not an applicability-domain outlier. All molecules showed leverage values below h^*. Therefore, no applicability-domain outlier was identified for the ln(η) model, and the model was interpreted within the structural space covered by the present decalin derivative library.

S4.3 Statistical analysis of the T_f regression model

The T_f model used the same descriptor set as the viscosity model to enable a consistent comparison of the structural variables affecting the two properties. The resulting regression equation for flash point is:

$$T_f = 206.240+0.863M-2.520I_{\alpha}-8.564I_{iso}+12.891I_{Cycle3}+11.828I_{Cycle4}+14.364I_{Cycle5}+15.521I_{Cycle6}+17.833I_{Cnor}-8.075I_{Fused}-5.228I_{Spiro}$$

The regression coefficients, multicollinearity diagnostics, model-performance metrics, residual analysis, outlier identification, and leverage-based applicability-domain assessment are summarized in Tables S4.4–S4.5 and Figure S4.2.

Table S4.4 Regression coefficients and multicollinearity diagnostics of the T_f model.

Descriptor	β	Standardized β	Std. Error	t-value	p-value	VIF
Intercept	206.240	—	7.697	26.795	<0.001	—
M	0.863	0.865	0.041	21.230	<0.001	5.380
I_{α}	-2.520	-0.057	1.079	-2.335	0.025	1.990
I_{iso}	-8.564	-0.141	1.832	-4.674	<0.001	1.755
I_{Cycle3}	12.891	0.189	2.178	5.918	<0.001	1.844
I_{Cycle4}	11.828	0.173	2.157	5.484	<0.001	1.916
I_{Cycle5}	14.364	0.210	2.282	6.294	<0.001	1.995
I_{Cycle6}	15.521	0.227	2.532	6.130	<0.001	2.081
I_{Cnor}	17.833	0.261	2.820	6.323	<0.001	2.159
I_{Fused}	-8.075	-0.145	2.051	-3.937	<0.001	2.023
I_{Spiro}	-5.228	-0.094	1.799	-2.906	0.006	2.006

Table S4.5 Statistical performance of the T_f model.

Metric	Value
R^2	0.977
Adjusted R^2	0.971
RMSE	3.370
MAE	2.214
Q^2	0.960
F-statistic	165.560
p-value	<0.001

The flash point model exhibits strong statistical performance, with $R^2 = 0.977$, adjusted $R^2 = 0.971$, $Q^2 = 0.960$, and RMSE = 3.370 K. The maximum VIF value is 5.380 for M, indicating no severe multicollinearity. Similar to the ln(η) model, the slightly higher VIF of M reflects its expected correlation with molecular size and structural class, whereas the ring-type and topology descriptors remain within a low-to-moderate VIF range.

Residual analysis, outlier identification, and leverage-based applicability-domain assessment were further performed for the T_f model. Because the same descriptor set was used, p = 10, n = 50, and the warning leverage threshold was:

$$h^* = \frac{3(p+1)}{n} = \frac{3(10+1)}{50} = 0.660 \quad \text{(S40)}$$

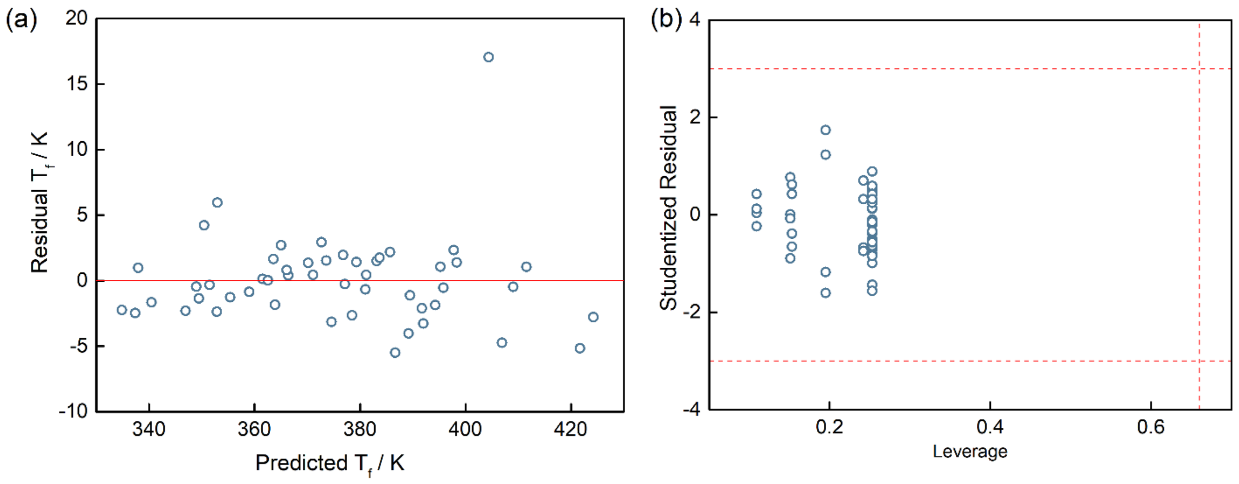

Figure S4.2. Residual and applicability-domain analysis of the T_f regression model.

(a) Residuals versus predicted T_f values. (b) Williams plot based on studentized residuals and leverage values. The vertical dashed line indicates the warning leverage threshold $h^* = 0.660$, and the horizontal dashed lines indicate studentized residuals of ±3.

As shown in Figure S4.2, most residuals are distributed around zero. Molecules with $|r_i|>3$ were treated as potential response outliers, whereas molecules with $h_i>h^*$ were considered potentially outside the applicability domain. The largest studentized residual was observed for α-S-Cnor (r_i = 5.170, h_i = 0.253), indicating that α-S-Cnor is a potential response outlier for the T_f model. However, its leverage value is much lower than h^* = 0.660, indicating that α-S-Cnor is not an applicability-domain outlier. All molecules showed leverage values below h^*. Therefore, no applicability-domain outlier was identified for the T_f model, and the model was interpreted within the structural space covered by the present decalin derivative library.

S5 Justification and robustness analysis of the multi-objective screening

S5.1 Screening procedure

The final screening was conducted in two steps. First, flash point and viscosity at −40 °C were used as feasibility constraints. Molecules satisfying $T_f \geq 360$ K and $\eta(-40 \circ C) \leq 16$ mPa·s were retained as feasible candidates. Second, the retained molecules were compared in terms of density, gravimetric NHOC, volumetric NHOC, and specific impulse. For the four-property comparison within each retained set, a molecule was considered non-dominated when no other retained molecule showed equal or higher values in density, gravimetric NHOC, volumetric NHOC, and I_{sp}, with at least one of these properties being higher. The screening based on the calculated

property values is described in Sections S5.1 and S5.2, whereas the influence of prediction uncertainty on the pass/fail classification is evaluated separately in Section S5.3.

S5.2 Sensitivity to the screening thresholds

Table S5.1 Distribution-based interpretation of the screening boundaries.

Property	Dataset used	Min	25% boundary	Median	75% boundary	Max	Selected boundary
T_f / K	Full molecular library	332.61	359.60	375.73	388.62	421.43	360
η at -40 °C / mPa·s	Molecules with $T_f \geq 360$ K	10.04	15.87	21.49	29.72	79.49	16

The $T_f \geq 360$ K boundary approximately removes the lowest 25% flash-point values in the full molecular library. After this flash-point pre-screening, the $\eta \leq 16$ mPa·s boundary approximately retains the lowest-viscosity 25% of the flash-point-qualified molecules. Therefore, these two values were used as engineering-oriented screening boundaries rather than absolute fuel specifications.

Among the ten feasible candidates, α-S-Cycle4 ranks first in density, gravimetric NHOC, volumetric NHOC, and specific impulse simultaneously. Therefore, α-S-Cycle4 is the only non-dominated candidate when the retained molecules are compared using these four-performance metrics.

Table S5.2 Sensitivity analysis of the viscosity and flash-point screening boundaries.

Screening condition	Candidate number	Non-dominated candidate(s) in the four-property comparison	Interpretation
$\eta \leq 14$ mPa·s, $T_f \geq 360$ K	4	α-S-Cycle4	Under a stricter viscosity condition, α-S-Cycle4 remains the only non-dominated candidate.
$\eta \leq 16$ mPa·s, $T_f \geq 360$ K	10	α-S-Cycle4	Main screening window; α-S-Cycle4 ranks first in all four-performance metrics.
$\eta \leq 18$ mPa·s, $T_f \geq 360$ K	12	α-S-Cycle4; α-L-Cycle3	With moderately relaxed viscosity, α-S-Cycle4 remains in the leading set, while α-L-Cycle3 enters because of its higher gravimetric NHOC and specific impulse.
$\eta \leq 16$ mPa·s, $T_f \geq 355$ K	12	α-S-Cycle4	With moderately relaxed flash-point screening, α-S-Cycle4 remains the only non-dominated candidate.

$\eta \le 16$ mPa·s, $T_f \ge 365$ K	7	β-S-Cycle4	Under a stricter flash-point condition, β-S-Cycle4 becomes the leading candidate, indicating that the S-Cycle4 motif remains competitive.
$\eta \le 16$ mPa·s, $T_f \ge 370$ K	3	α-n-C4; β-iso-C5	Further increasing the flash-point requirement shifts the retained set toward higher-flash-point alkyl-substituted molecules.
$\eta \le 16$ mPa·s, $T_f \ge 380$ K	0	None	This condition is overly restrictive for the low-viscosity screening objective.
$\eta \le 25$ mPa·s, $T_f \ge 360$ K	22	α-S-Cycle4; α-L-Cycle4; β-F-Cycle6	A relaxed viscosity condition allows more viscous high-energy candidates and changes the design priority.
$\eta \le 25$mPa·s, $T_f \ge 380$ K	7	α-L-Cycle4; β-F-Cycle6	This represents a high-flash-point and relaxed-viscosity screening case, which is different from the main low-viscosity screening objective.

The sensitivity analysis indicates that the exact selected molecule depends on the adopted low-temperature fluidity and safety requirements. However, α-S-Cycle4 remains the leading candidate under the main screening window and moderate variations of the viscosity or flash-point limits. When the flash-point requirement is increased to $T_f \ge 365$ K, β-S-Cycle4 becomes the leading candidate. Therefore, the exact selected isomer may change under stricter safety requirements, but the S-Cycle4 structural motif remains robust and highly competitive within the practical screening window. This analysis addresses sensitivity to the choice of screening boundaries; prediction uncertainty at fixed boundaries is considered separately in Section S5.3.

S5.3 Uncertainty-aware screening based on validation errors

Prediction uncertainty was incorporated into the feasibility screening using the validation errors obtained in Section S1.10. Because the viscosity constraint was defined at 233.15 K, the RMSE obtained from the ten-point validation subset at this temperature (2.267 mPa·s) was used as the viscosity error scale. The corresponding RMSE for flash point was 1.853 K. Although negative mean signed errors were observed for both properties, symmetric one-RMSE ranges were adopted here so that the uncertainty classification did not depend on the direction of the observed bias.

Under the principal screening criteria of $T_f \ge 360$ K and $\eta(-40°C) \le 16$ mPa·s, the candidates were first classified using their calculated property values. Prediction uncertainty was then considered using one-RMSE adverse or favorable shifts according to the initial classification. The classification criteria are summarized in Table S5.3.

Table S5.3 Classification criteria used in the uncertainty-aware screening

Initial status	One-RMSE assessment	Uncertainty-aware classification	Interpretation
Retained	η+RMSE$_\eta$ ≤ 16 mPa·s and T_f-RMSE$_{Tf}$ ≥ 360 K	Robustly feasible	Both constraints remain satisfied under the adverse-error scenario.
Retained	Eitherη+RMSE$_\eta$ > 16 mPa·s or Tf-RMSE$_{Tf}$< 360 K	Boundary-sensitive	At least one constraint may be crossed within the one-RMSE range.
Excluded	η-RMSE$_\eta$ ≤ 16 mPa·s and Tf+RMSE$_{Tf}$≥ 360 K	Boundary-sensitive	The favorable-error range overlaps the feasible region.
Excluded	Either η-RMSE$_\eta$ > 16 mPa·s or Tf+RMSE$_{Tf}$< 360 K	Robustly excluded	The candidate remains outside the feasible region even under the favorable-error scenario.

On this basis, the ten candidates retained by the original screening and the four initially excluded candidates whose favorable one-RMSE ranges overlap the feasible region were further examined, as summarized in Table S5.4.

Table S5.4 Uncertainty-aware classification of candidates

Molecule	η / mPa·s	One-RMSE η range / mPa·s	T_f / K	One-RMSE (T_f) range / K	Initial status	Uncertainty-aware classification
β-n-C3	10.041	7.774–12.308	361.658	359.805–363.511	Retained	Boundary-sensitive
α-n-C4	11.999	9.732–14.266	371.519	369.666–373.372	Retained	Robustly feasible
α-iso-C4	13.5	11.233–15.767	362.539	360.686–364.392	Retained	Robustly feasible
β-n-C4	14.633	12.366–16.900	375.131	373.278–376.984	Retained	Boundary-sensitive

β-iso-C4	15.873	13.606–18.140	367.742	365.889–369.595	Retained	Boundary-sensitive
β-iso-C5	15.553	13.286–17.820	376.848	374.995–378.701	Retained	Boundary-sensitive
α-S-Cycle4	13.326	11.059–15.593	362.04	360.187–363.893	Retained	Robustly feasible
β-S-Cycle4	15.348	13.081–17.615	366.802	364.949–368.655	Retained	Boundary-sensitive
α-F-Cycle5	14.296	12.029–16.563	365.213	363.360–367.066	Retained	Boundary-sensitive
β-F-Cycle5	15.231	12.964–17.498	366.888	365.035–368.741	Retained	Boundary-sensitive
α-n-C3	9.165	6.898–11.432	358.149	356.296–360.002	Excluded	Boundary-sensitive
β-iso-C3	11.64	9.373–13.907	358.912	357.059–360.765	Excluded	Boundary-sensitive
α-iso-C5	17.723	15.456–19.990	371.444	369.591–373.297	Excluded	Boundary-sensitive
α-L-Cycle3	17.913	15.646–20.180	371.523	369.670–373.376	Excluded	Boundary-sensitive

Among the ten candidates initially retained by the screening, α-n-C4, α-iso-C4, and α-S-Cycle4 remain feasible over the full one-RMSE adverse-error range and are therefore classified as robustly feasible. The other seven retained candidates are boundary-sensitive. Four initially excluded molecules—α-n-C3, β-iso-C3, α-iso-C5, and α-L-Cycle3—also have one-RMSE ranges that overlap the feasible region, whereas the remaining excluded molecules remain outside the screening window.

For α-S-Cycle4, the one-RMSE adverse values are 15.593 mPa·s for viscosity and 360.187 K for flash point, both remaining within the corresponding limits. Among the three robustly feasible candidates, α-S-Cycle4 also retains the highest calculated density, gravimetric NHOC, volumetric NHOC, and specific impulse. Prediction uncertainty therefore affects the membership of the feasible set but does not change the identification of α-S-Cycle4 as the best-balanced candidate.

References

1. Chi, H.; Li, G.; Guo, Y.; Xu, L.; Fang, W., Excess molar volume along with viscosity, flash point, and refractive index for binary mixtures of cis-decalin or trans-decalin with C9 to C11 n-alkanes. *Journal of Chemical & Engineering Data* **2013,** 58, (8), 2224-2232.

2. Outcalt, S. L.; Laesecke, A., Measurements of density and speed of sound of JP-10 and a comparison to rocket propellants and jet fuels. *Energy & fuels* **2011,** 25, (3), 1132-1139.

3. Tardajos, G.; Diaz Pena, M.; Lainez, A.; Aicart, E., Speed of sound in and isothermal compressibility and isobaric expansivity of pure liquids at 298.15 K. *Journal of Chemical and Engineering Data* **1986,** 31, (4), 492-493.

4. Laesecke, A.; Outcalt, S. L.; Brumback, K. J., Density and speed of sound measurements of methyl-and propylcyclohexane. *Energy & fuels* **2008,** 22, (4), 2629-2636.

5. Li, D.; Wang, J.; Gao, Y.; Zhan, X.; Li, M.; Wang, Y., Density, viscosity, and refractive index of binary mixtures of fatty acid ethyl esters with ethylcyclohexane. *Journal of Chemical & Engineering Data* **2019,** 64, (12), 5324-5331.

6. Luning Prak, D. J.; Cowart, J. S.; Trulove, P. C., Density and Viscosity from 293.15 to 373.15 K, Speed of Sound and Bulk Modulus from 293.15 to 343.15 K, Surface Tension, and Flash Point of Binary Mixtures of Bicyclohexyl and 1,2,3,4-Tetrahydronaphthalene or Trans-decahydronaphthalene at 0.1 MPa. *Journal of Chemical & Engineering Data* **2016,** 61, (1), 650-661.

7. Luning Prak, D. J.; Lee, B. G., Density, Viscosity, Speed of Sound, Bulk Modulus, Surface Tension, and Flash Point of Binary Mixtures of 1,2,3,4-Tetrahydronaphthalene and Trans-decahydronaphthalene. *Journal of Chemical & Engineering Data* **2016,** 61, (7), 2371-2379.

8. Bai, S. D.; Dumbacher, P.; Cole, J. W., Development of Advanced Hydrocarbon Fuels at Marshall Space Flight Center. **2002**.

9. Qin, X.; Cao, X.; Guo, Y.; Xu, L.; Hu, S.; Fang, W., Density, viscosity, surface tension, and refractive index for binary mixtures of 1, 3-dimethyladamantane with four C10 alkanes. *Journal of Chemical & Engineering Data* **2014,** 59, (3), 775-783.

10. Speros, D. M.; Rossini, F. D., Heats of Combustion and Formation of Naphthalene, the Two Methylnaphthalenes, cis and trans-Decahydronaphthalene, and Related COMPOUNDS1. *The Journal of Physical Chemistry* **1960,** 64, (11), 1723-1727.

11. Smith, N.; Good, W., Enthalpies of combustion of ramjet fuels. *AIAA Journal* **1979,** 17, (8), 905-907.

12. Prosen, E.; Johnson, W.; Rossini, F., Heats of formation and combustion of the normal alkylcyclopentanes and cyclohexanes and the increment per CH2 group for several homologous series of hydrocarbons. *Journal of Research of the National Bureau of Standards* **1946,** 37, (1), 51-56.

13. Good, W.; Moore, R.; Osborn, A.; Douslin, D., The enthalpies of formation of ethylcyclobutane, methylenecyclobutane, and 1, 1-dimethylcyclopropane. *The Journal of Chemical Thermodynamics* **1974,** 6, (3), 303-310.

14. Kong, W.-H.; Li, Y.-W.; Wang, J.-B.; Li, X.-Y., Theoretical study of hydrogen abstraction from quadricyclane by small radicals. *Computational and Theoretical Chemistry* **2021,** 1200, 113232.

15. Montgomery, R. L.; Rossini, F. D.; Mansson, M., Enthalpies of combustion, vaporization, and formation of phenylbenzene, cyclohexylbenzene, and cyclohexylcyclohexane; enthalpy of hydrogenation of certain aromatic systems. *Journal of Chemical and Engineering Data* **1978,** 23, (2), 125-129.

16. Steele, W. V.; Watt, I., The standard enthalpies of formation of adamantanoid compounds 4. Methyladamantanes. *The Journal of Chemical Thermodynamics* **1977,** 9, (9), 843-849.

17. Thomas, J.; Marcia, L.; Arno, L.; Eric, W.; Richard, A., Thermochemical and thermophysical properties of JP-10. *NISTIR* **2006,** 6640, 18-21.

18. Assael, M. J.; Dalaouti, N. K., Measurement of the viscosity of cyclopentane from 210 to 310 K and pressures up to 25 MPa. *High Temperatures-High Pressures* **2000,** 32, (2), 179-184.

19. Wang, H.; Zhang, B.; Gong, S.; Liu, Y.; Liu, G., Experimental and theoretical study on 2-ethylnorbornane pyrolysis under atmospheric and high pressure. *Fuel* **2020,** 272, 117724.

20. Rudakova, M. A.; Zarezin, D. P.; Shorunov, S. V.; Samoilov, V. O.; Ilyin, S. O.; Maximov, A. L.; Bermeshev, M. V., High-Energy-Density Liquid Spiro-Norbornanes from Methylenenorbornane. *Energy & Fuels* **2022,** 36, (19), 11930-11939.

21. Woodroffe, J.-D.; Zhang, D. D.; Harvey, B. G., Diels–Alder Cycloaddition of Cyclopentadiene with α-Olefins for the Synthesis of High-Performance Sustainable Aviation Fuels. *Energy & Fuels* **2024,** 38, (19), 18763-18768.

22. Xing, Y.; Shao, D.; Fang, W.; Guo, Y.; Lin, R., Vapor pressures and flash points for binary mixtures of tricyclo [5.2. 1.02. 6] decane and dimethyl carbonate. *Fluid phase equilibria* **2009,** 284, (1), 14-18.

23. Saldana, D. A.; Starck, L.; Mougin, P.; Rousseau, B.; Pidol, L.; Jeuland, N.; Creton, B., Flash Point and Cetane Number Predictions for Fuel Compounds Using Quantitative Structure Property Relationship (QSPR) Methods. *Energy Fuels* **2015,** 25, (9), 3900-3908.
